# Repurposing a clinically approved prescription Colquhounia root tablet to treat diabetic kidney disease via suppressing PI3K/AKT/NF-kB activation

**DOI:** 10.1186/s13020-021-00563-7

**Published:** 2022-01-04

**Authors:** Zhaochen Ma, Yudong Liu, Congchong Li, Yanqiong Zhang, Na Lin

**Affiliations:** Key Laboratory of Beijing for Identification and Safety Evaluation of Chinese Medicine, Institute of Chinese Materia Medica, China Academy of Chinese Medical Sciences, No. 16, Nanxiaojie, Dongzhimennei, Beijing, 100700 China

**Keywords:** *Tripterygium wilfordii* Hook F-based therapeutics, Colquhounia root tablet, Diabetic kidney disease, Imbalance of immune-inflammation system, Network pharmacology

## Abstract

**Background:**

Growing clinical evidences show the potentials of Colquhounia root tablet (CRT) in alleviating diabetic kidney disease (DKD). However, its pharmacological properties and underlying mechanisms remain unclear.

**Methods:**

‘Drug target-Disease gene’ interaction network was constructed and the candidate network targets were screened through evaluating node genes' topological importance. Then, a DKD rat model induced by high-fat diet/streptozotocin was established and used to determine pharmacological effects and network regulatory mechanisms of CRT against DKD, which were also verified using HK2 cell model induced by high glucose.

**Results:**

The candidate network targets of CRT against DKD were involved into various type II diabetes-related and nephropathy-related pathways. Due to the topological importance of the candidate network targets and the important role of the imbalance between immunity and inflammation in the pathogenesis of DKD, PI3K/AKT/NF-кB signaling-mediated immune-modulatory and anti-inflammatory actions of CRT were selected to be experimentally verified. On the basis of high-fat diet (HFD) / streptozotocin (STZ)-induced DKD rat model, CRT effectively reduced the elevated level of blood glucose, decreased the accumulation of renal lipid, suppressed inflammation and the generation of ECM proteins, and ameliorated kidney function and the renal histopathology through inhibiting the activation of PI3K, AKT and NF-кB proteins, reducing the nuclear accumulation of NF-кB protein and the serum levels of downstream cytokines, which were in line with the in vitro findings.

**Conclusions:**

Our data suggest that CRT may be the promising candidate drug for treating DKD via reversing the imbalance of immune-inflammation system mediated by the PI3K/AKT/NF-кB/IL-1β/TNF-α signaling.

**Supplementary Information:**

The online version contains supplementary material available at 10.1186/s13020-021-00563-7.

## Background

Diabetic kidney disease (DKD) is a common and severe microvascular complication caused by diabetes. According to the United States Renal Data System, DKD accounts for up to 50% of end-stage renal disease (ESRD) cases [1, 2], and also the main risk factor for death of patients with diabetes [3, 4]. It is characterized by hyperglycemia, persistent proteinuria, hypertension and impaired renal function [5–8]. The main therapeutic strategy of the early DKD is hypoglycemic and antihypertensive combined symptomatic treatment, and the main therapeutic strategy of the late DKD is kidney transplantation. Unfortunately, the efficacy of the current therapeutic strategies is not very satisfying [9]. Although DKD was traditionally indicated as a non-inflammatory glomerular disease caused by metabolic and hemodynamic changes, growing clinical and experimental evidences show that the imbalance between immunity and inflammation may also play an important role in the pathogenesis of DKD [9]. However, there are still few clinical trials on immune-modulatory and/or anti-inflammatory therapeutics [10–12].

*Tripterygium wilfordii* Hook F. (*Tw*HF) has significant anti-inflammatory and immunosuppressive effects, and is used in the treatment of a wide spectrum of autoimmune disorders and inflammatory diseases with favorable therapeutic efficacy for a long time in China [13, 14]. Among various *Tw*HF-based therapeutics, Colquhounia root tablets (CRT) has been approved by National Medical Products Administration for the treatment of rheumatoid arthritis and systemic lupus erythematosus because of its anti-inflammatory, immunosuppressive and other hormone-like effects. Growing clinical evidences based on relative small samples also show the potentials of CRT in alleviating DKD [15–17]. Especially, Li et al. reported that the clinical effective rate of CRT in the treatment of 70 cases of DKD with massive albuminuria was 94.3% [18].

Since the scientific evidences confirming the efficacy of CRT in treating DKD is still inadequate and the necessity of exploring new drugs to attenuate renal inflammation, the aim of the current study was to investigate the main pharmacological properties and the underlying molecular mechanisms of CRT against DKD using an integrated network-based computational and experimental strategy.

## Materials and methods

### Data collection and network investigation

#### Prediction of CRT putative targets

The chemical components containing CRT were collected from our TCM-related database, ETCM (http://www.tcmip.cn/ETCM/index.php/Home/Index/, Released in 2018) [14]. Then, we chose the chemical components with moderate to good drug-likeness by calculating the quantitative estimate of drug-likeness (QED) score based on models in the Pipeline Pilot ADMET collection as the candidate bioactive components of CRT (QED > 0.49) for the following target predictions. The putative targets hit the candidate bioactive components of CRT were predicted using MedChem Studio (version 3.0; SimulationsPlus, Lancaster, CA, USA, 2012) according to the structural and functional similarities of drugs. The component-putative target pairs with similarity scores higher than 0.80 (high similarity) were selected for further investigation.

#### Collection of DKD-related genes

Known therapeutic targets of DKD were collected from the DrugBank database (http://www.drugbank.ca/, version: 5.1.8). Only disease genes that belong to human genes in FDA-approved DKD therapeutic drug targets can be included. In addition, the DKD-related genes were also collected from HPO (The Human Phenotype Ontology, https://hpo.jax.org/app/, last updated on June 8, 2021) and DisGeNet (http://www.disgenet.org/, version: 7.0), by searching clinical symptoms of DKD and extracting the corresponding gene sets. Finally, all the genes obtained from the above three databases were merged and the redundancy was removed.

#### Functional enrichment analyses

Functional enrichment analyses of CRT putative targets were performed using the DAVID (Database for Annotation, Visualization and Integrated Discovery, https://david.ncifcrf.gov/tools.jsp, version: 6.7) based on the HPO data and the KEGG pathway data (Kyoto Encyclopedia of Genes and Genomes, http://www.genome.jp/kegg/, last updated on October 16, 2012). Only clinical symptoms and KEGG pathways with p values < 0.05 (corrected using the Bonferroni method) were selected.

#### "Disease Gene-Drug Target" interaction network construction and analysis

"Disease Gene-Drug Target" interaction network was constructed using links between DKD-related genes and CRT putative targets according to the public database STRING (Search Tool for Known and Predicted Protein–Protein Interactions, http://string-db.org/, version: 10.0). The gene–gene interactions with combined scores higher than 0.7 were selected in this study. Then, the three topological features, including the node degree, betweenness and closeness were calculated to screen the network target with the topological importance in the networks. The nodes with high interconnections within the network were divided into different functional modules by using the Markov clustering algorithm. The networks were visualized by Cytoscape software (version: 3.8.0).

### In vivo experimental validation

#### Ethics statement

The in vivo experimental validation with animals were performed in the Institute of Chinese Materia Medica, China Academy of Chinese Medical Sciences, Beijing, China. All experimental protocols were approved by the Research Ethics Committee of the Institute of Chinese Materia Medica (No. IACUC-G16045). Animal experiments were carried out in accordance with the guidelines and regulations for the care and use of laboratory animals of the Center for Laboratory Animal Care, China Academy of Chinese Medical Sciences, Beijing, China.

#### Animals and treatment

Male Sprague–Dawley (SD) rats (n = 48, 190–210 g in weight) were purchased from Guangdong Medical Laboratory Animal Center (production license no. SCXK 2013-0002, Guangzhou, China). All rats were kept under specific pathogen-free conditions, with a constant temperature of 24± 1 °C in a 12-h light/12-h dark cycle room and adlibitum water and food access. Prior to the experiments, the rats were allowed a 1-week acclimatization period. A total of 48 SD rats were randomly divided into normal control group (n = 8) and model group (n = 40) and provided adlibitum access to standard rodent chow and HFD, respectively. The model was established with HFD (60% energy from fat, 20% carbohydrate and 20% protein) (# D12492, Research Diets, Inc., NJ, USA). After 4 weeks of dietary manipulation, HFD-fed rats were injected intraperitoneally with 35 mg/kg Streptozotocin (STZ, freshly dissolved in precooling citrate buffer solution, pH 4.5, #V900890, Sigma-Aldrich, St. Louis, MO, USA) twice a week. A week after STZ injection, rats with blood glucose levels over 11.1 mmol/L were divided into the DKD group (n = 8), Metformin (#PHR1084, Sigma-Aldrich, St. Louis, MO, USA) group (135 mg/kg, n = 8), CRT (Pharmaceutical Factory of the Chongqing Academy of Chinese Materia Medica, Chongqing, China) low-dosage group (CRT-L, 145 mg/kg, n = 8), CRT medium-dosage group (CRT-M, 290 mg/kg, n = 8), CRT high-dosage group (CRT-H, 580 mg/kg, n = 8). The normal control group rats (CON) were injected with the solvent of STZ (precooling citrate buffer solution, pH 4.5). In the course of administration, the rats in the model group and the treatment group were fed with HFD until the end of the study, which was 9 weeks. The DKD rat model had a mortality rate of about 20%. At the end of the experiments, the rats in different groups were all anesthetized with pentobarbital sodium, the blood was taken from the abdominal aorta, and the rats were killed by cervical dislocation. Finally, all the rats were dissected and samples were taken.

#### Detection of biochemical indicators

Body weight, blood glucose and urine volume of the rats were measured once a week. Blood samples were taken once a week to detect the levels of insulin, C-peptide and glycosylated hemoglobin. The experiment was terminated at the end of 63 days and the rats were fasted for 12 h. Then, the urine was collected by metabolic cage, and the microalbuminuria, creatinine, and 24 h urine protein were detected by ELISA. The levels of IL-1β, TNF-α, total cholesterol (TC), triglyceride (TG), low density lipoprotein (LDL-C), and high density lipoprotein (HDL-C) in serum, and the levels of IL-1β, TNF-α, Collagen l and Fibronectin in cell culture medium were all measured by ELISA kits. Detailed information of all ELISA kits is provided in Additional file 1: Table S1.

#### Histopathological evaluation

Renal tissues obtained from different groups were stained with H & E, periodic acid- Schiff (PAS), MASSON, and periodic acid silver methenamine (PASM) staining to observe the histopathological changes. The methods were the same as our previous studies or manufacturer’s instructions [19].

#### Immunohistochemical staining

Immunohistochemical staining was conducted using an UltraSensitive SP (rabbit/mouse) immunohistochemistry (IHC) kit (# KIT-9706, KIT-9701, KIT-9709; MX Biotechnologies, Fuzhou, China), which contained endogenous peroxidase blocking solution, serum, secondary antibody, streptavidin-peroxidase, and diaminobenzidine (DAB) substrate-chromogen (DAB-0031, MX Biotechnologies, Fuzhou, China). The Collagen l (dilution 1:800, #PAB46098, Bio-Swamp, Wuhan, China) and Fibronectin (dilution 1:800, # PAB46097, Bio-Swamp, Wuhan, China) antibodies were used. The rest of the experimental methods, the intensity of dyeing score and quantitative methods were the same as our previous studies [19].

#### Immunofluorescence assay

Immunofluorescence analyses were also carried out according to our previous description [19]. The antibodies were NF-кB (#14,220–1-AP, Proteintech, IL, USA) and p-NF-кB (#YP0191, Immuno Way, TX, USA).

### In vitro experimental validation

The Human proximal tubular epithelial cell line (HK-2) (# GDC0152, China Center for Type Culture Collection, Wuhan, China) was used for in vitro experimental validation. HK-2 cells were maintained in Dulbecco’s modified Eagle medium media (DMEM, #SH30022-01B, Thermo Scientific/HyClone, USA) supplemented with low glucose (5.6 mmol/L) and 10% fetal bovine serum (FBS) in a humidified 5% CO_2_ incubator at 37 ℃. Trypsin was used to digest the HK-2 cells after being grown to 80–90%. Moreover, the cells were added to the 6-well plate with the density of 1.5 × 10^5^ cells per well. After the cells were adhered to the wall, the medium containing 0.5% serum was replaced and cultured for 24 h, so that the cells were in a resting phase. Then, HK-2 cells were divided into the following groups: (1) control group (CON, D-glucose 5.6 mmol/L), (2) high glucose group (HG, D-glucose 30 mmol/L), (3) CRT low-dosage group (CRT-L, 10 µg/mL), (4) CRT high-dosage group (CRT-H, CRT 50 µg/mL), (5) Dapagliflozin group (DAP, 10 µmol/L). All treatment groups were cultured in high glucose medium (D-glucose 30 mmol/L). After 48 h of coculture, the supernatant and cells were collected for further analysis.

### Western blot analysis

Protein expression levels of PI3K, p-PI3K, AKT, p-AKT, NFkB and p-NF-кB (p65) in renal tissues and cells of different groups were detected by western blot analysis according to our previous studies [19–21]. The detailed information about the antibodies is as follows: PI3K (#4249, CST, MA, USA), p-PI3K (#bs-3332R, Bioss Inc, Beijing, China), AKT (#ab89402, abcam, MA, USA), p-AKT (#ab81283, abcam, MA, USA), NF-кB (p65) (#YM3111, Immuno Way, TX, USA), p-NF-кB (p65) (#YP0191, Immuno Way, TX, USA), and GAPDH (# 2118, CST, MA, USA).

### Statistical analyses

All experiments in the current study were performed in triplicate. Statistical analyses were performed using GraphPad Prism (version 7.0). Data are shown as the mean ± SD, and were analyzed by one-way ANOVA followed by a least significant difference (LSD) test. P values < 0.05 were considered to be significant.

## Results

### Pharmacological potentials of CRT against DKD

A total of 672 putative target genes of CRT were predicted as summarized in Additional file 1: Table S2. Then, we collected 3472 DKD-related genes from HPO, DisGeNet and Drugbank databases (Additional file 1: Table S3), of which 390 were putative targets of CRT. According to the enrichment analysis, the putative targets of CRT were significantly associated with several clinical symptoms of DKD, such as pollakiuria, diabetic neuropathy, inflammation, diabetic retinopathy and hypertensive disease, etc*.* (all p < 0.05, Additional file 1: Table S4). In terms of functional modules, the putative targets of CRT were involved into immune system regulation, substance metabolism, energy synthesis and decomposition, as well as hormone synthesis and metabolism (all p < 0.05, Additional file 1: Table S5). The above data imply that CRT might have potentials to reverse the pathological changes of DKD, which promoted us to explore its pharmacological mechanisms.

## CRT alleviates DKD mainly by reversing the immune-inflammatory imbalance

**Fig. 1:**
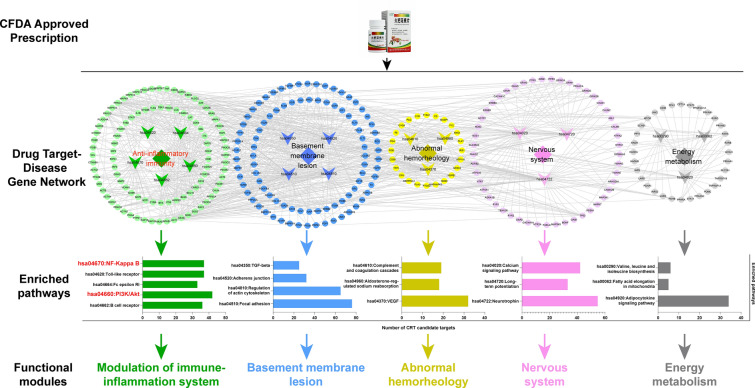
Network-based analysis illustrates that CRT may alleviate DKD mainly by reversing the immune-inflammatory imbalance. In the network, the circular nodes represent the candidate targets of CRT against DKD, the arrow nodes represent the pathways, and the diamond nodes represent the functional modules

“Disease Gene-Drug Target” interaction network was constructed with 23,923 links among 561 CRT targets and 2851 DKD-related genes. After calculating the network topological features, we identified a total of 231 candidate targets of CRT against DKD (Additional file 1: Table S6), which were significantly enriched in five functional modules, including the regulation of immune-inflammatory response, alleviation of renal basement membrane lesions, regulation of abnormal renal hemorheology, regulation of nervous system and energy metabolism (Fig. 1 and Additional file 1: Table S7). Notably, the regulation of immune-inflammatory module was the most enriched functional module by the candidate targets of CRT. Therefore, we would like to perform in vivo and in vitro experiments to verify the immune modulatory and anti-inflammatory actions.

### CRT effectively reduces blood glucose, insulin and alleviates lipid metabolic disturbance in DKD rats

**Fig. 2:**
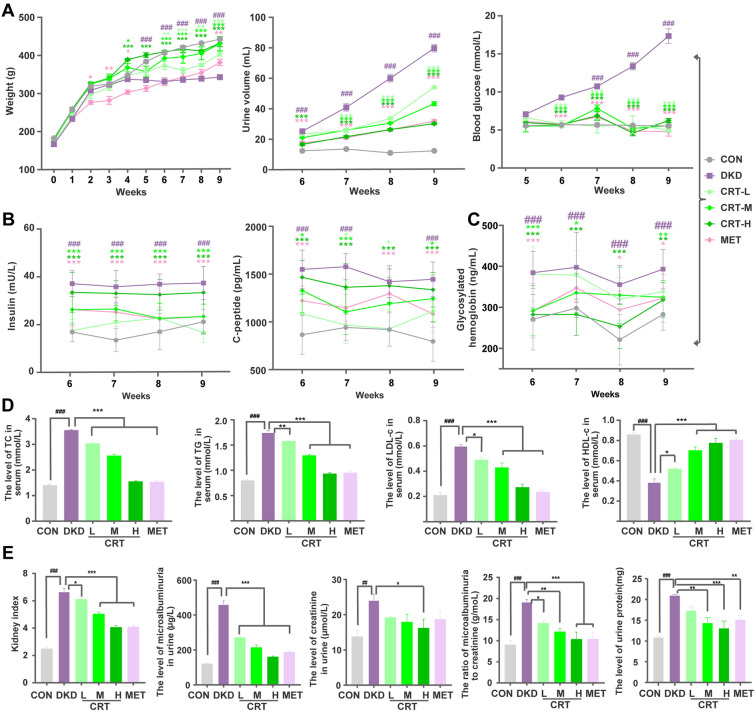
Dynamic changes of body weight, urine volume, blood glucose and insulin-related index, as well as the levels of index closely related to lipid metabolism, renal function and inflammation status in DKD rats. **A** Effects of CRT on body weight, urine volume, blood glucose of DKD rats. **B**–**D** Levels of **B** Insulin, C-peptide, **C** Glycosylated hemoglobin, **D** TC, TG, LDL-c and HDL-c in serum of DKD rats. **E** Kidney index, the level of urinary microalbuminuria, Creatinine and their ratio, urine protein in urine. Data are shown as the mean ± SD. ^#^p < 0.05, ^##^p < 0.01, ^###^p < 0.001, comparison with the CON group; *p < 0.05, **p < 0.01, ***p < 0.001, comparison with the DKD group

Compared with the normal control rats, DKD rats induced by HFD/STZ were featured as the reduced body weights, increased urine volume and blood glucose, which were effectively reversed by the administration of CRT at 2 weeks (Fig. 2A). In addition, the significant elevation in the serum levels of insulin and C-peptide were observed in DKD rats from 6 to 9 weeks, and fell back to normal after the administration of CRT with high dose (Fig. 2B). Glycosylated hemoglobin may accurately reflect the degree of long-term blood glucose control, and may be the gold standard for blood glucose control in patients with diabetes [22]. As shown in Fig. 2C, a significant increase in glycosylated hemoglobin level was observed in the DKD rats, which was reversed by the administration of CRT. It is considered that the accumulation of free lipids in glomeruli and tubules leads to renal lipotoxicity, which in turn leads to tubulointerstitial fibrosis [23]. We further detected the serum levels of lipid metabolism indicators in different groups, the administration of CRT with various dosages significantly reduced the serum levels of TC, TG, LDL-C and elevated that of HDL-C (Fig. 2D).

### CRT effectively improves renal function, extracellular matrix deposition and ameliorates inflammatory reaction in DKD rats and HK-2 cells

**Fig. 3:**
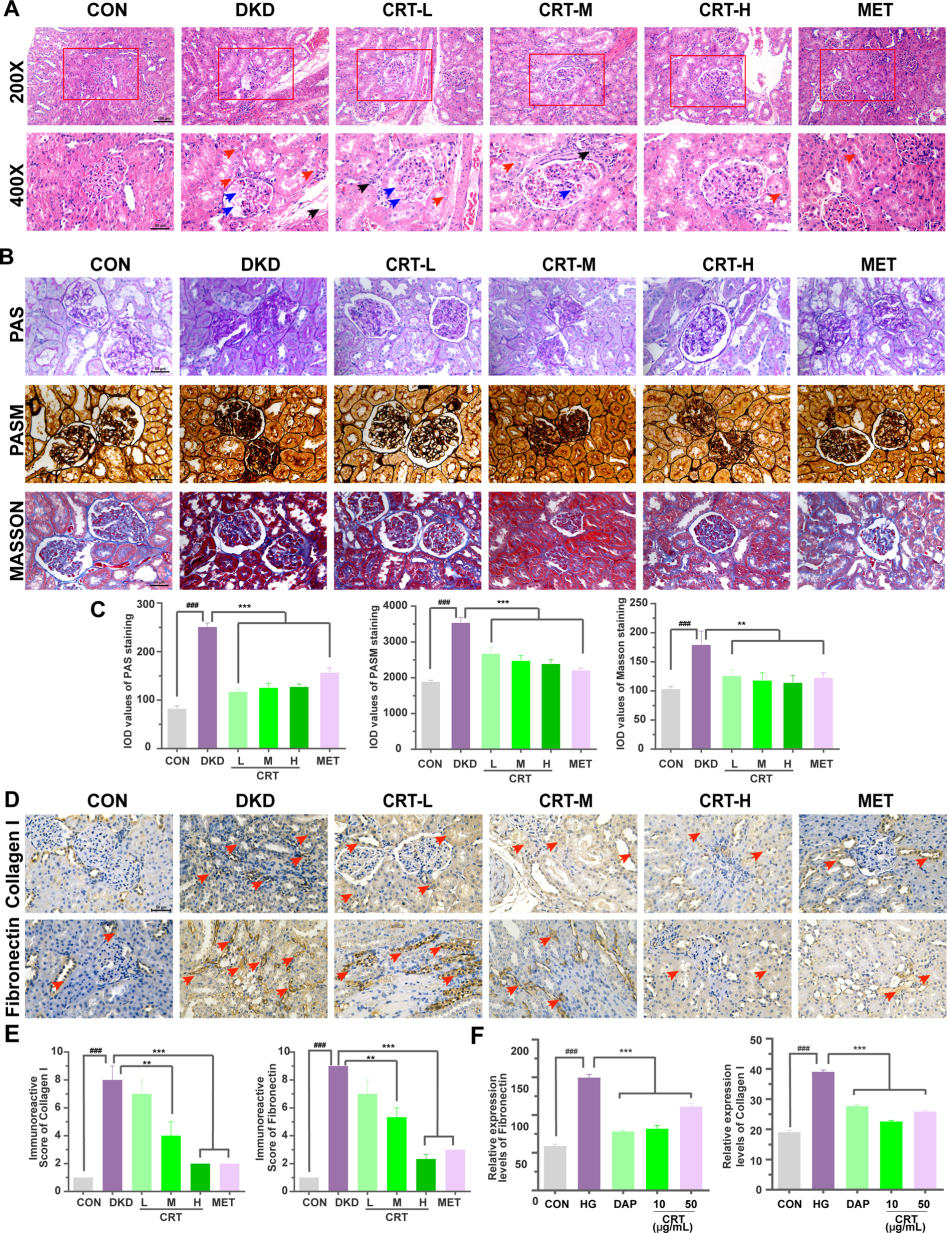
Effects of CRT on the histopathological changes of kidney examined by H & E, PAS, PASM, MASSON stainings, and on the expression patterns of the ECM markers in kidney tissues of DKD rats detected by immunohistochemistry. **A** Representative images of the kidney tissues in different groups examined by H & E staining. Original magnification, × 200, 400. Scale bars represent 100, 50 µm. Red boxes mark the areas with typical pathological changes. **B** Representative images of the kidney tissues in different groups examined by PAS, PASM, MASSON stainings. Original magnification, × 400. Scale bars represent 50 µm. **C** Quantitative analyses of PAS, PASM and Masson stainings. **D** Expression patterns of Collagen I and Fibronectin in the kidney tissues of different groups examined by immunohistochemistry. Original magnification, × 400. Scale bars represent 50 µm. **E** Semiquantitative data of immunoreactivities of fibronectin and collagen I proteins in the kidney tissues of different groups, respectively. **F** Expression levels of fibronectin and collagen I proteins in HK-2 cells treated by high glucose detected by ELISA. Data are shown as the mean ± SD. ^#^p < 0.05, ^##^p < 0.01, ^###^p < 0.001, comparison with the CON group; ^*^p < 0.05, ^**^p < 0.01, ^***^p < 0.001, comparison with the DKD group

As shown in Fig. 2E, DKD rats exhibited the elevated kidney index, 24 h urine protein, urinary microalbuminuria and creatinine levels, and the ratio of urinary albumin to creatinine (UACR), which were all significantly decreased following the administration of CRT with high dosage. Regarding to the histopathological changes of the kidney tissues in different groups, Fig. 3A showed the lobulated glomeruli, severely atrophied glomerular vessels (blue arrow), and vacuolar degeneration cytoplasm (red arrow), inflammatory infiltration (black arrow) in DKD rats. In addition, PAS staining showed that the mesangial matrix of kidneys was significantly increased. Consistently, PASM staining of glomeruli revealed the slightly thickened basement membrane, and Masson staining revealed the slightly increased interstitial fibrosis (Fig. 3B, C). In contrast, the administration of CRT with high dose for 4 weeks effectively ameliorated the histopathological changes in diabetic kidney [24, 25]. Similar results were found in MET treatment group. All these results indicate that CRT may improve the deterioration of renal function and structural changes in DKD rats.

Since the alterations of extracellular matrix (ECM) are one of the key pathological modifications of DKD [26], we also examined the expression patterns of profibrotic molecules fibronectin and collagen I proteins using immunohistochemistry. As shown in Fig. 3D, E, the administration of CRT significantly attenuated the enhanced immunoreactive intensity and reduced the increased number of positive immunoreactive cells of both fibronectin and collagen I in DKD rats (all P < 0.05). Consistently, the expression of fibronectin and collagen I in HK-2 cells treated by high glucose showed the same trend (Fig. 3F). Moreover, the serum levels of TNF-α and IL-1β in DKD rats were significantly higher than the normal rats, but were markedly reduced in the drug treatment group. Consistently, the expression of TNF-α and IL-1β in HK-2 cells treated by high glucose showed the same trend (Fig. 5A, B).

### CRT inhibits the activation of PI3K/AKT/NF-кB signaling in vivo and in vitro

**Fig. 4:**
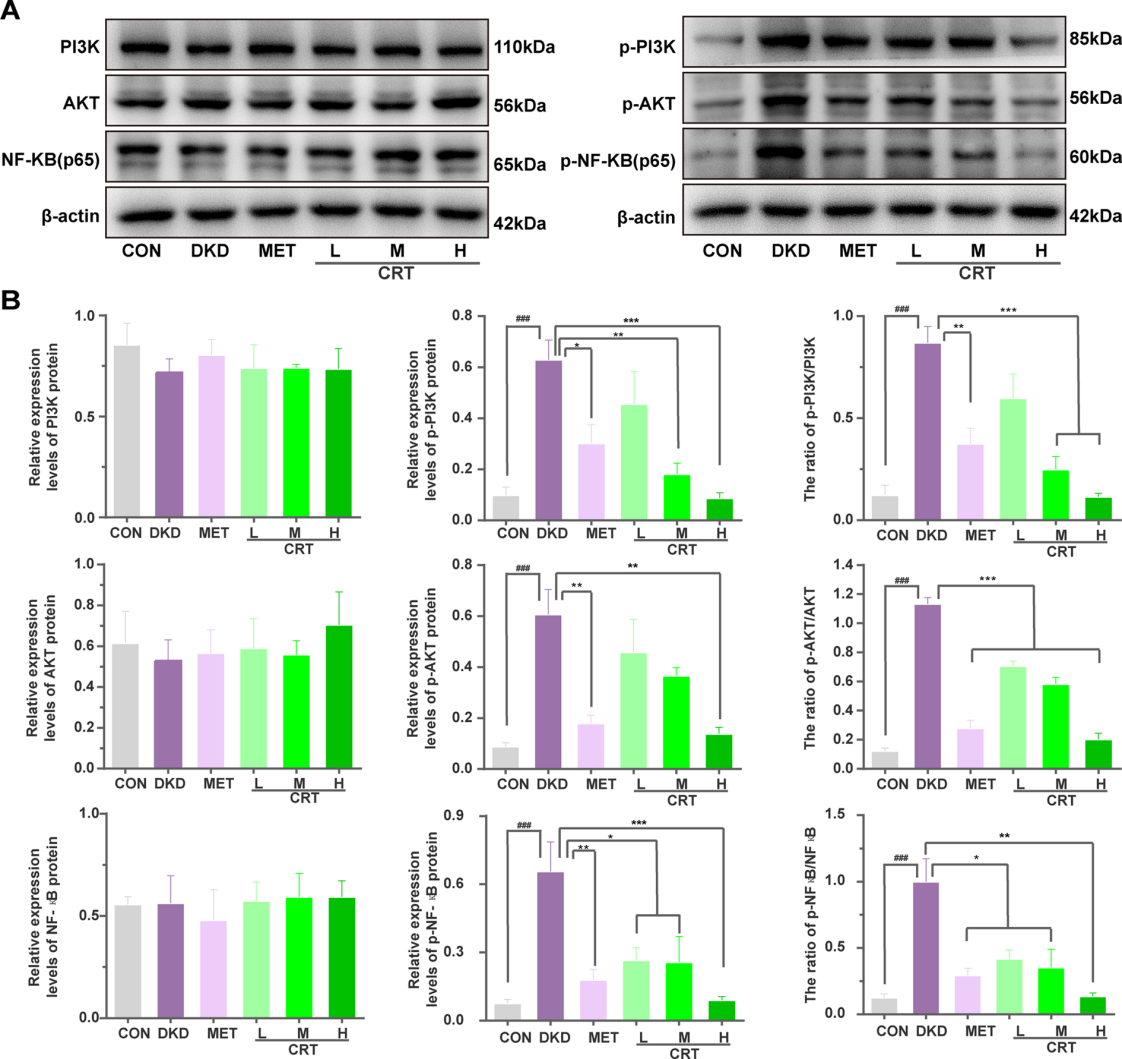

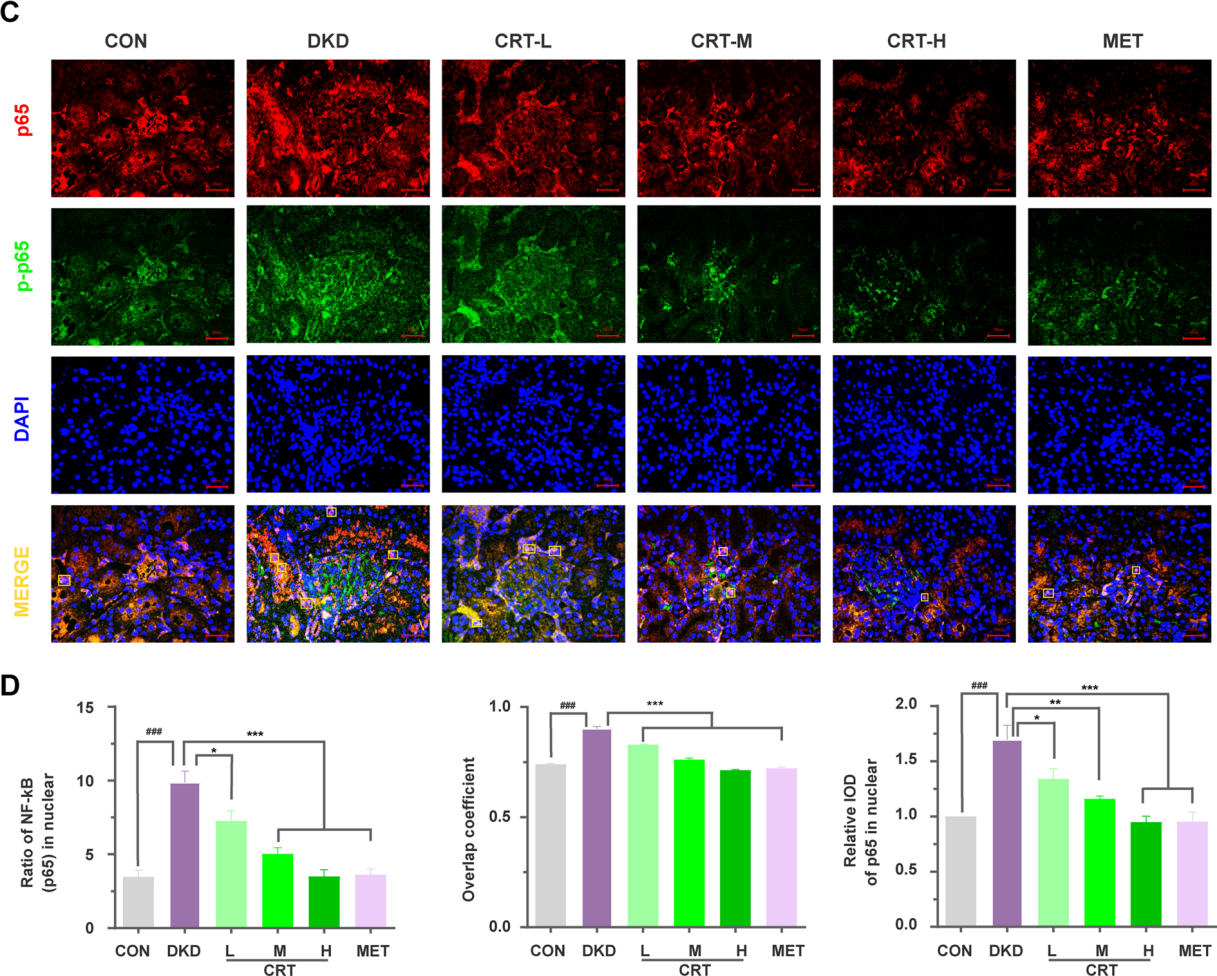
Effects of CRT on the expression levels of CRT candidate targets involved in PI3K/AKT/NF-кB signaling in kidney tissues of DKD rats. **A** Representative western blots of various target proteins. **B** Relative protein expression levels of PI3K, p-PI3K, the ratio of p-PI3K to PI3K, AKT, p-AKT, the ratio of p-AKT to AKT, NF-кB (p65), p-NF-кB (p65) and the ratio of p-NF-кB (p65) to NF-кB (p65), respectively. **C** Expression and distribution of NF-кB (p65) protein in renal tissues of DKD rats. Original magnification, × 400. **D** Ratio of the number of cells with NF-кB (p65) nuclei translocation to the total number of cells, overlap coefficient and relative IOD value of NF-кB (p65) in nuclear in renal tissues cells of DKD rats. Data are shown as the mean ± SD. ^#^p < 0.05, ^##^p < 0.01, ^###^p < 0.001, comparison with the CON group; ^*^p < 0.05, ^**^p < 0.01, ^***^p < 0.001, comparison with the DKD group

**Fig. 5:**
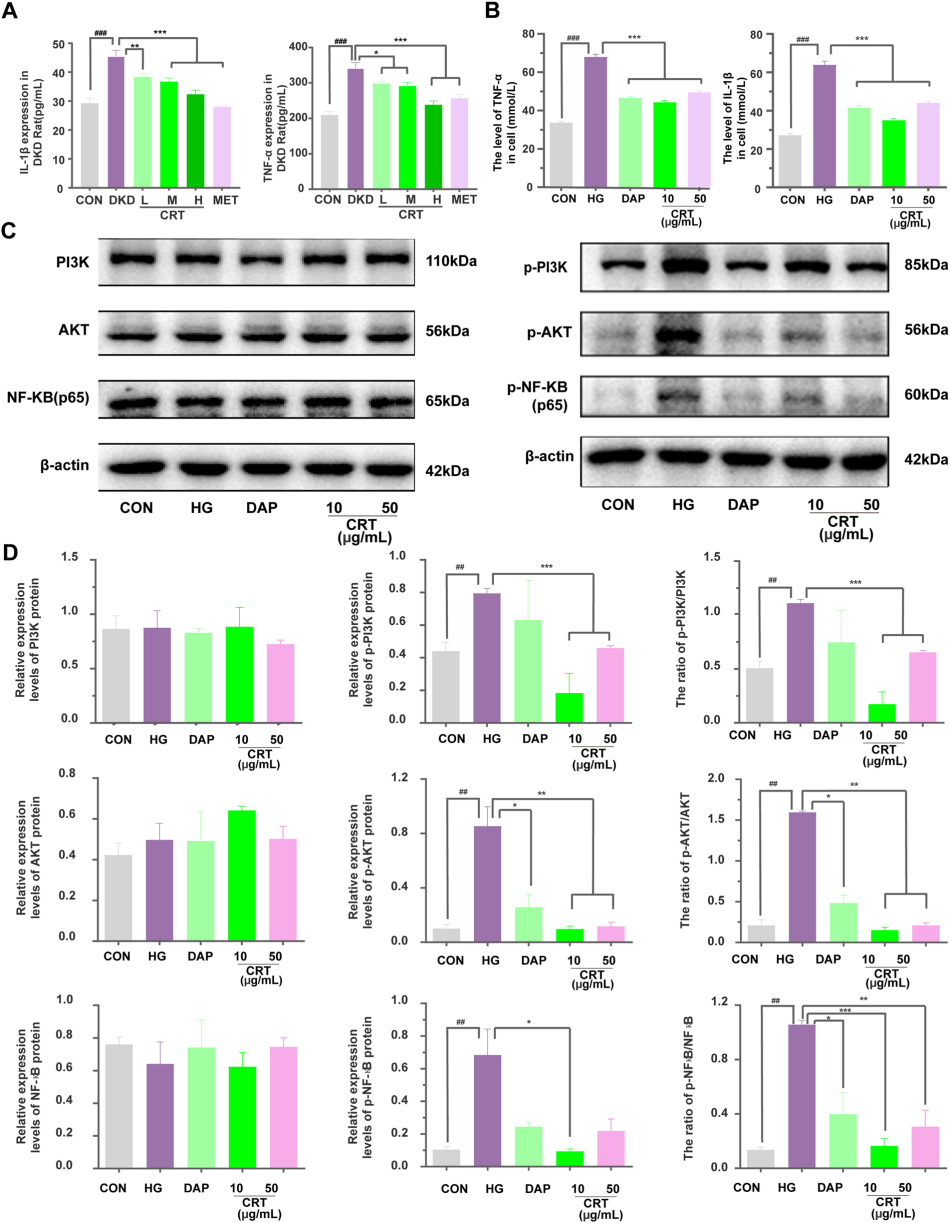
Effects of CRT on the expression levels of CRT candidate targets involved in PI3K/AKT/NF-кB signaling, and the distribution of NF-кB (p65) protein in HK-2 cells treated with High Glucose. **A**, **B** Expression levels of IL-1β and TNF-α in serum and HK-2 cells treated with high glucose detected by ELISA. **C** Representative western blots. **D** Relative protein expression levels of PI3K, p-PI3K, the ratio of p-PI3K to PI3K, AKT, p-AKT, the ratio of p-AKT to AKT, NF-кB (p65), p-NF-кB (p65), the ratio of p-NF-кB (p65) to NF-кB (p65) in HK-2 cells treated with High Glucose. Data are shown as the mean ± SD. ^#^p < 0.05, ^##^p < 0.01, ^###^p < 0.001, comparison with the CON group; *p < 0.05, **p < 0.01, ***p < 0.001, comparison with the HG group

**Fig. 6:**
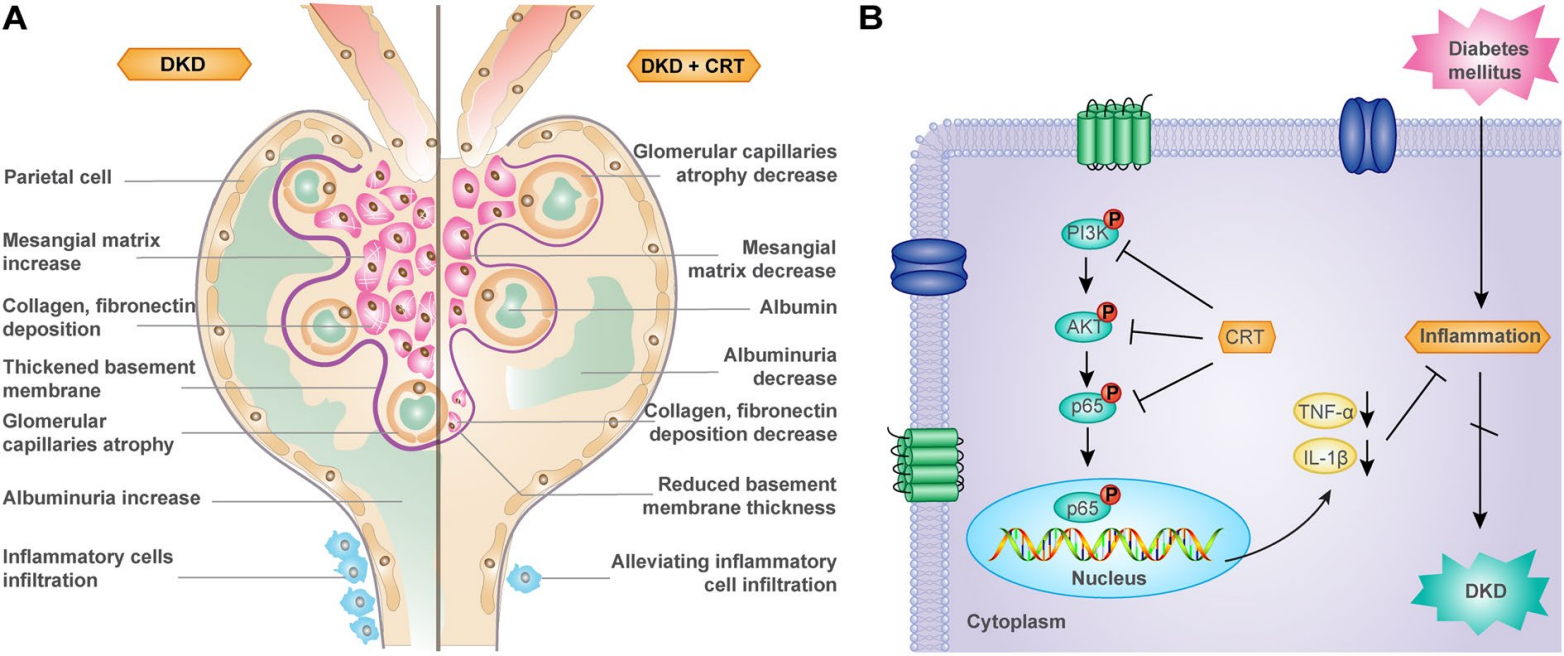
Illustration of the pharmacological mechanisms underlying the therapeutic effects of CRT against DKD. **A** Histopathological changes of DKD kidney before and after the administration of CRT. **B** The mechanism of CRT—induced inhibition of inflammatory cytokine production

According to our network analysis, the major network targets of CRT against DKD were all significantly enriched into PI3K/AKT/NF-кB pathway (Fig. 1), which may play a pivotal role in regulating inflammatory response of various diseases. Consistently, the results of our western blot analyses showed that the expression of p-PI3K, p-AKT, and p-NF-кB proteins, as well as the ratios of p-PI3K/total PI3K, p-AKT/total AKT and p-NF-кB/total NF-кB in renal tissues of DKD rats and high glucose-induced HK-2 cells were significantly increased comparing with the normal controls. In contrast, CRT effectively reduced the expression levels of p-PI3K, p-AKT and p-NF-кB proteins, as well as the ratio of phosphorylation to total proteins both in vivo (Fig. 4A, B) and in vitro (Figs. 5C, D and 6). Similarly, our immunofluorescence assay further showed the marked NF-кB (p65) translocation into the nucleus in renal tissues of DKD rats, and the situation was reversed after the administration of CRT (Fig. 4C, D).

## Discussions

The immune-inflammation imbalance has been indicated to promote the occurrence and the progression of DKD, implying that drugs with immune-modulatory and/or anti-inflammatory actions may be promising candidates for the treatment of this disease [27]. However, clinical trials of anti-inflammatory agents with favorable short-term outcomes are only just beginning to be published, and it will take a long time for the large-scale use of the corresponding drugs [9]. *Tw*HF is used in the treatment of a wide spectrum of inflammatory and autoimmune diseases due to its conspicuous anti-inflammatory and immunosuppressive effects [13, 20, 21]. In the current study, we focused on the *TwHF*-based Chinese patent prescription CRT, which have been indicated to exert good immunomodulatory and anti-inflammatory effects in previous studies [28]. Following a series of computational analyses on “Disease Gene-Drug Target” interaction network, our data firstly showed that the candidate network targets of CRT against DKD might be significantly associated with pathways related to immune-inflammation and remission of renal basement membrane lesion. Both the key network target identification and functional module analysis revealed that the inflammation-immune mediated by PI3K/AKT/NF-kB signal may be associated with the pharmacological properties of CRT to ameliorate DKD. Experimentally, CRT effectively reduced the elevated level of blood glucose, ameliorated kidney function and the renal histopathology abnormality, including inflammation, abnormal lipid metabolism, and glomerular structural damages in HFD/STZ-induced DKD rats. Furthermore, the above efficacy of CRT is closely related to the activation of PI3K, AKT and NF-kB proteins, the reduction of nuclear accumulation of NF-kB protein, and the serum levels of downstream cytokines, such as IL-1β and TNF-α, which were in line with the in vitro findings based on HK-2 cells induced by high glucose. These findings highlight the main pharmacological properties of CRT against DKD by reversing the imbalance of immune-inflammation system during the occurrence and development of this disease.

The pathogenesis of DKD involves inflammation, fibrosis and abnormal lipid metabolism [9]. Persistent hyperglycemia destroys the glomerular filtration barrier, leading to glomerular structural damage, resulting in urinary protein or albumin leakage, and which, in turn further aggravates the progression of DKD [29]. Mesangial matrix expansion and thickening of the glomerular basement membrane are the main hallmarks of DKD [30]. Both inflammatory cytokines and high glucose can stimulate tubular epithelial cells to produce collagen I and fibronectin, which are the markers of ECM. Excessive accumulation of ECM in mesangium and glomerular basement membrane may accelerate tubulointerstitial fibrosis [31, 32]. Consistently, our *in vivo* experiments observed the enhanced albuminuria, urinary protein excretion (UACR), aggravated mesangial matrix expansion, basement membrane thickening and extracellular matrix deposition in glomerular, and worsening renal tissue inflammation and interstitial fibrosis, as well as elevated lipid metabolism in DKD rats. In contrast, the administration of CRT effectively reversed the above pathological changes and attenuated the development of DKD. Chronic inflammation and fibrosis play important roles in the occurrence and development of complications of DKD [25]. Like renal interstitial fibrosis, DKD may be stimulated by persistent hyperglycemia and inflammatory factor [33]. It has been reported that TNF-α and IL-1β may induce proximal tubular epithelial cells to secrete ECM markers (type I collagen and fibronectin), which accelerates renal tubular interstitial fibrosis [34].

NF-kB signaling is the key pathway to tissue inflammation, because NF-kB may control the expression of inflammatory cytokines, chemokines and adhesion molecules, all of which play a key role in controlling inflammation [20, 35–38]. In addition, the inhibition of NF-kB may effectively alleviate renal inflammation and fibrosis caused by persistent hyperglycemia in DKD [25]. In the current study, the activation of NF-kB protein and the levels of TNF-α and IL-1β were positively associated with the renal inflammatory damage and extracellular matrix deposition in both DKD rats and HK-2 cells treated by high glucose, which were also consistent with previous reports [25]. Moreover, network analysis results showed that the upstream signaling of inflammation such as PI3K/Akt, Toll-like receptor 4/2, Tim 3, HMBG1, and so on, may be the important mechanism by which CRT alleviates inflammatory damage in DKD. Recent evidence show that PI3K/Akt signaling pathways may be critical in the modulation of ECM expression and tubule-interstitial fibrosis in chronic renal diseases. Even though, whether PI3K/Akt signaling may be associated with the anti-inflammatory effects of CRT is still unclear. Herein, we focused on the upstream signaling of NF-kB signaling-mediated inflammatory pathway, PI3K/Akt signaling, and found that the persistent hyperglycemia activated PI3K/Akt pathway, which promoted the activation of NF-кB signaling and the release of inflammatory cytokines, subsequently leading to massive proteinuria, tubulointerstitial inflammatory infiltration, mesangial matrix expansion and basement membrane thickening in DKD rats. On the contrary, CRT efficiently reversed the above pathological changes by suppressing the activation of PI3K/AKT/NF-kB pathway and inhibiting the translocation of p65 into the nucleus.

## Conclusion

Our findings provide the evidences that the *Tw*HF-based prescription, CRT may be the promising candidate drugs for the treatment of DKD via reversing the imbalance of Immune-Inflammation system which is mediated by PI3K/AKT/NF-кB/IL-1β/TNF-α signaling during the disease progression.

## Supplementary Information

**Additional file 1: Table S1.** Includes detailed information of all enzyme-linked immunosorbent assay kits. **Table S2.** Includes 672 putative target genes of CRT predicted using ETCM database and TCMIP v2.0 platform. **Table S3.** Includes 3472 DKD-related genes from Human Phenotype Ontology (HPO), DisGeNet and Drugbank databases. **Table S4.** Includes enrichment results of clinical symptoms of 390 CRT putative targets. **Table S5. **Includes enrichment results of functional modules of 390 CRT putative targets. **Table S6.** Includes 231 candidate targets of CRT against DKD in “Disease Gene-Drug Target” interaction networks based on calculated network topological features. **Table S7.** Includes enrichment results of functional modules of 231 CRT candidate targets.

## Data Availability

All supporting data are included within the main article and its Additional files.

## Abbreviations

ADMET: Absorption distribution metabolism excretion toxicity
ANOVA: Analysis of variance
AKT: Protein kinase B
CFDA: National medical products administration
CON: Control
CRT: Colquhounia root tablets
CRT-L: Colquhounia root tablets—low-dosage group
CRT-M: Colquhounia root tablets—medium-dosage group
CRT-H: Colquhounia root tablets—high-dosage group
DAB: Diaminobenzidine
DAP: Dapagliflozin
DAVID: Database for annotation, visualization andintegrated discovery
DKD: Diabetic kidney disease
ESRD: End stage renal disease
ELISA: Enzyme linked immunosorbent assay
ETCM: Encyclopedia of traditional chinese medicine
FDA: Food and drug administration
GAPDH: Glyceraldehyde-3-phosphate dehydrogenase
HDL-C: High density lipoprotein—cholesterol
H & E: Hematoxylin and eosin
HFD: High fat diet
HK-2: Human kidney-2
HPO: Human phenotype ontology
IHC: Immunohistochemistry
IL-1β: Interleukin-1β
KEGG: Kyoto encyclopedia of genes and genomes
LDL-C: Low density lipoprotein—cholesterol
LSD: Least significant difference
NF-кB: Nuclear factor—kappa B
PAS: Periodic acid- schiff
PASM: Periodic acid-silver metheramine
PI3K: Phosphoinositide 3-kinase
QED: Quantitative estimate of drug-likeness
SD: Sprague dawley
STZ: Streptozotocin
TC: Total cholesterol
TCM: Traditional Chinese medicine
TG: Triglyceride
TNF-α: Tumour necrosis factor-α
*Tw*HF: *tripterygium wilfordii* Hook f
