# Supplementary material for: Repurposing a clinically approved prescription Colquhounia root tablet to treat diabetic kidney disease via suppressing PI3K/AKT/NF-kB activation
Source: Chin Med. 2022 Jan 4;17:2. doi: 10.1186/s13020-021-00563-7 (PMC8725443; doi:10.1186/s13020-021-00563-7)
Supplement: Supplementary file 1 — Additional file 1: Table S1. Includes detailed information of all enzyme-linked immunosorbent assay kits. Table S2. Includes 672 putative target genes of CRT predicted using ETCM database and TCMIP v2.0 platform. Table S3. Includes 3472 DKD-related genes from Human Phenotype Ontology (HPO), DisGeNet and Drugbank databases. Table S4. Includes enrichment results of clinical symptoms of 390 CRT putative targets. Table S5. Includes enrichment results of functional modules of 390 CRT putative targets. Table S6. Includes 231 candidate targets of CRT against DKD in “Disease Gene-Drug Target” interaction networks based on calculated network topological features. Table S7. Includes enrichment results of functional modules of 231 CRT candidate targets. [file 13020_2021_563_MOESM1_ESM.pdf]

Additional File 1

Table S1. Detailed information of all enzyme-linked immunosorbent assay kits.

Table S2. A list of 672 putative target genes of CRT predicted using ETCM database and TCMIP v2.0 platform.

Table S3. A list of 3472 DKD-related genes collected from Human Phenotype Ontology (HPO), DisGeNet and Drugbank databases.

Table S4. Enrichment results of clinical symptoms of 390 CRT putative targets.

Table S5. Enrichment results of functional modules of 390 CRT putative targets.

Table S6. A list of 231 candidate targets of CRT against DKD in “Disease Gene-Drug Target” interaction networks based on calculated network topological features.

Table S7. Enrichment results of functional modules of 231 CRT candidate targets.

Table S1 Detailed information of all enzyme-linked immunosorbent assay kits

Kit Name	Company	Cat. No.
Rat Interleukin-1 beta (IL-1β) ELISA Kit	Wuhan Purity Biological Technology Co., LTD, Wuhan, China (http://chundubio.com/)	CD-108059-ELISA
Rat Tumor Necrosis Factor (TNF-α) ELISA Kit		CD-108075-ELISA
Rat C-Peptide (C-P) ELISA Kit		CD-106837-EA
Rat Insulin ELISA (INS) Kit		CD-108763-ELISA
Rat Glycosylated hemoglobin (GHb) ELISA Kit		CD-107198-ELISA
Rat Total cholesterol (TC) ELISA Kit		CD-108177-ELISA
Rat Triglyceride (TG) ELISA Kit		CD-107224-ELISA
Rat High density liptein cholesterol (HDL-c) ELISA Kit		CD-109024-ELISA
Rat Low density liptein cholesterol (LDL-c) ELISA Kit		CD-109021-ELISA
Rat Urinary microalbuminuria (mAlb) ELISA Kit		CD-108123-ELISA
Rat Creatinine (Cr) ELISA Kit		CD-107048-EA

Table S2 A list of 672 putative target genes of CRT predicted using ETCM database and TCMIP v2.0 platform

Gene symbol	Gene symbol	Gene symbol	Gene symbol	Gene symbol	Gene symbol
ABAT	CEBPA	FABP6	IMPDH2	PDE8B	SCN5A
ABCB1	CEBPB	FADS1	INSR	PDE9A	SCN7A
ABCB4	CES1	FADS2	ITGAL	PDHB	SCN8A
ABCG2	CHCHD4	FASN	ITGB1BP1	PGR	SCN9A
ACADM	CHD1	FBLN1	ITGB2	PHGDH	SDS
ACADSB	CHD8	FCGR1A	ITPR1	PHYH	SDSL
ACAT1	CHGA	FECH	ITPR2	PIK3CA	SEC14L2
ACE	CHRFAM7A	FFAR1	ITPR3	PIK3CB	SEC14L3
ACE2	CHRM1	FGF1	JAK1	PIK3CD	SEC14L4
ACHE	CHRM2	FGF16	JUN	PIK3CG	SEC14L6
ACSL3	CHRM3	FGF18	JUP	PIM1	SEMA5A
ACSL4	CHRM4	FGF2	KCNH2	PINK1	SERPINE1
ACTB	CHRM5	FGF4	KCNK1	PKLR	SERPINE2
ACY1	CHRNA1	FKBP1A	KCNK6	PKM	SF3B3
ADA	CHRNA10	FKBP1B	KCNN4	PKP2	SHANK3
ADAM8	CHRNA2	FOLR1	KDM5D	PKP3	SHBG
ADH1C	CHRNA3	FOLR2	KIF14	PLA2G1B	SHMT1
ADK	CHRNA4	FOLR3	KRT12	PLA2G2E	SHMT2
ADORA1	CHRNA5	FPGS	LARP1	PLAT	SI
ADORA2A	CHRNA6	FTCD	LCT	PLG	SIGMAR1
ADORA2B	CHRNA7	GAA	LHCGR	PLOD1	SLC16A1
ADORA3	CHRNA9	GABRA1	LIPF	PLOD2	SLC16A2
ADRA1A	CHRNB1	GABRA2	LPL	PLOD3	SLC16A3
ADRA1B	CHRNB2	GABRA3	LRAT	PNLIP	SLC16A4
ADRA1D	CHRNB3	GABRA4	MAF1	PON1	SLC16A5
ADRA2A	CHRNB4	GABRA5	MAP1A	PPARA	SLC16A6
ADRA2B	CHRND	GABRA6	MAP2	PPARD	SLC16A7
ADRA2C	CHRNE	GABRB1	MAP4	PPARG	SLC16A8
ADRB1	CHRNG	GABRB2	MAPK7	PPIA	SLC18A1
ADRB2	CHUK	GABRB3	MAPKAP1	PPIF	SLC18A2
ADRB3	CLCN2	GABRD	MAPT	PPP2CA	SLC23A1
AFM	CMPK1	GABRE	MET	PPP2CB	SLC2A1
AGTR1	CMPK2	GABRG1	MGAM	PPP2R1A	SLC2A2
AGXT2	CMYA5	GABRG2	MLNR	PPP3R2	SLC47A1
AHR	CNR1	GABRG3	MMP9	PRDX4	SLC5A2
AIP	CNR2	GABRP	MPL	PRDX5	SLC6A2
AKAP1	COL5A1	GABRQ	MPO	PRKCA	SLC6A3
AKAP6	COLQ	GART	MPST	PRKCB	SLC6A4
AKR1C1	COMT	GCH1	MTFMT	PRKCD	SLC6A5
AKR1C2	COX1	GCSH	MTHFD1	PRKCE	SLC6A9

Table S2 A list of 672 putative target genes of CRT predicted using ETCM database and TCMIP v2.0 platform

Gene symbol	Gene symbol	Gene symbol	Gene symbol	Gene symbol	Gene symbol
AKR1D1	COX2	GDF5	MTHFD1L	PRKCG	SLC7A11
AKT1	COX3	GFI1	MTHFD2	PRKCI	SLC8A1
AKT1S1	COX4I1	GGCX	MTHFD2L	PRKCQ	SLCO1B1
ALB	COX5A	GLDC	MTHFR	PRKCZ	SLCO1B3
ALDH1A1	COX5B	GLRA1	MTHFS	PRKD1	SMAD2
ALDH1A2	COX6A2	GLRA2	MTNR1A	PRKD2	SMAD6
ALDH1A3	COX6B1	GLRA3	MTNR1B	PRKDC	SMAD7
ALDH1L1	COX6C	GLRA4	MTOR	PRLR	SMO
ALDH1L2	COX7A1	GLRB	MTR	PRNP	SNX25
ALDH5A1	COX7B	GNRHR	MTTP	PRR5	SOAT1
ALDH9A1	COX7C	GP9	NCF1	PRR5L	SOAT2
ALKBH2	COX8A	GPER1	NCF2	PSAT1	SOCS1
ALKBH3	CPS1	GPHN	NCF4	PSPH	SPR
ALOX5	CREB1	GPRC5A	NCOA1	PTEN	SRD5A1
AMDHD1	CSNK2A1	GRIA2	NCOA2	PTGER1	SRD5A2
AMT	CSNK2B	GRIK2	NEU1	PTGER2	SRR
AMY2A	CTH	GRIN1	NEU2	PTGER3	STK17B
ANXA1	CXCL13	GRIN2A	NF2	PTGER4	SUPV3L1
AOC1	CYBA	GRIN2B	NKX3-1	PTGFR	TBC1D10C
APBB1	CYBB	GRIN2C	NLGN1	PTGIR	TBPL1
APOE	CYCS	GRIN2D	NOS2	PTGIS	TBXAS1
AR	CYGB	GRIN3A	NOTCH2	PTGS1	TELO2
ATIC	CYP11B2	GRIN3B	NOX1	PTGS2	TGFB1
ATM	CYP17A1	GRM7	NOXO1	PTK2B	TGFB2
ATP1A1	CYP19A1	GSK3B	NQO2	PTPN2	TGFB3
ATP1A2	CYP1B1	GSN	NR0B1	RAC1	TGFBR2
ATP1A3	CYP27B1	GSS	NR1H4	RAC2	THNSL2
ATP5A1	CYP3A4	GTF2H5	NR1I2	RAC3	TIGAR
ATP5B	CYP51A1	H2AFY	NR1I3	RARA	TLR4
ATP5C1	CYR61	HAL	NR3C1	RARB	TMLHE
B2M	DAPK2	HAS1	NR3C2	RARG	TOP1
BBOX1	DBH	HCK	NRXN1	RARRES1	TOP2A
BCHE	DDC	HDAC1	NT5C2	RBP1	TOP2B
BCL2	DEPTOR	HDAC2	NT5M	RBP3	TP53
BEND3	DGKA	HDAC4	OGDH	RDH11	TPMT
BGLAP	DHFR	HDAC5	OGFOD1	RDH12	TRPV1
BHMT	DHODH	HDAC9	OGFOD2	RDH13	TSC1
BHMT2	DHRS3	HIBCH	OPRD1	RDH14	TSPO

BRAF	DHRS4	HIF1A	OPRK1	RDH5	TSPYL2
BRF1	DHX33	HIPK2	OPRM1	RDH8	TTI1
CACNA1A	DIAPH1	HMGCR	OPTN	RDX	TTPA

Table S2 A list of 672 putative target genes of CRT predicted using ETCM database and TCMIP v2.0 platform

Gene symbol	Gene symbol	Gene symbol	Gene symbol	Gene symbol	Gene symbol
CACNA1B	DLG4	HOXA5	ORM1	RELN	TUBA1A
CACNA1C	DNMT1	HPGD	ORM2	REST	TUBA1B
CACNA1D	DPYD	HRH1	P2RY12	RETSAT	TUBA1C
CACNA1F	DPYS	HRH2	P3H1	RICTOR	TUBA3C
CACNA1G	DRD1	HRH4	P3H2	RIPK1	TUBA3D
CACNA1H	DRD2	HSD11B1	P3H3	RIPK3	TUBA3E
CACNA1I	DRD3	HSD11B2	P4HA1	RLBP1	TUBA4A
CACNA1S	DRD4	HSD17B1	P4HTM	RPL23A	TUBA8
CACNA2D1	DRD5	HSD3B1	PADI4	RPTOR	TUBB
CACNA2D2	DTYMK	HSD3B2	PAH	RTN2	TUBD1
CACNA2D3	DUT	HSP90AA1	PAM	RUVBL2	TUBE1
CACNB1	DYRK2	HSPA2	PC	RXRA	TUBG1
CACNB2	EBP	HSPB1	PDE10A	RXRB	TUBG2
CACNB3	EGLN1	HTR1A	PDE11A	RXRG	TYMS
CACNB4	EGLN2	HTR1B	PDE1A	RYR1	TYR
CACNG1	EGLN3	HTR1D	PDE1B	S100A1	UBA1
CAD	EHHADH	HTR2A	PDE1C	S100A12	UCN
CALR	EIF3F	HTR2B	PDE2A	S100A13	UGT3A1
CALY	ELL	HTR2C	PDE3A	S100A2	UMPS
CAMLG	ELOVL4	HTR3A	PDE3B	S100A9	UROC1
CASP1	ENG	HTR4	PDE4A	S100B	VDR
CASP3	ENPP1	HTR6	PDE4B	SCN10A	VEGFA
CASQ1	ERBB2	HYAL2	PDE4C	SCN11A	VTI1A
CAV3	ERO1A	ICE1	PDE4D	SCN1A	WDHD1
CBR1	ERO1B	ICE2	PDE5A	SCN1B	WFS1
CBS	ESR1	IFNG	PDE6A	SCN2A	WLS
CCL24	ESR2	IGHG2	PDE6B	SCN2B	WNT3A
CCL3	ESRRA	IKBKB	PDE6C	SCN3A	WNT4
CD300A	ESRRB	IL1B	PDE7A	SCN3B	ZC3H8
CDK6	ESRRG	IL4	PDE7B	SCN4A	
CDKN1A	F12	IMPDH1	PDE8A	SCN4B	

Table S3 A list of 3472 DKD-related genes

Gene symbol	Gene symbol	Gene symbol	Gene symbol	Gene symbol	Gene symbol
AAAS	CHRNB1	FSHB	LACC1	PFAS	SLC36A1
AADAT	CHRNB2	FSHR	LAG3	PFKM	SLC36A2
AARS	CHRNB3	FST	LAGE3	PFN1	SLC37A4
AARS2	CHRNB4	FTCD	LAMA2	PGAM2	SLC38A3
AASS	CHRND	FTL	LAMA3	PGAP2	SLC38A8
ABAT	CHRNE	FTO	LAMA4	PGAP3	SLC39A14
ABCA1	CHRNG	FUCA1	LAMB2	PGF	SLC39A4
ABCA12	CHST14	FUS	LAMB3	PGK1	SLC3A1
ABCA3	CHST3	FUZ	LAMC2	PGM1	SLC40A1
ABCA4	CHUK	FXN	LAMP1	PGM3	SLC44A1
ABCA7	CIB2	FXYD2	LAMP2	PHB	SLC44A2
ABCB1	CIDEC	FZD4	LARGE1	PHEX	SLC44A3
ABCB11	CIITA	G6PC	LARP7	PHF21A	SLC44A4
ABCB4	CISD2	G6PC3	LARS	PHF6	SLC45A2
ABCB6	CKAP2L	G6PD	LARS2	PHGDH	SLC46A1
ABCB7	CLCF1	GAA	LAS1L	PHKA1	SLC47A1
ABCC1	CLCN1	GAB1	LAT	PHKA2	SLC4A1
ABCC10	CLCN2	GABBR1	LBR	PHKB	SLC4A11
ABCC2	CLCN5	GABBR2	LCA5	PHKG2	SLC4A4
ABCC3	CLCN7	GABRA1	LCAT	PHOSPHO1	SLC52A1
ABCC4	CLCNKA	GABRA2	LCK	PHOX2A	SLC52A2
ABCC5	CLCNKB	GABRA3	LCN2	PHOX2B	SLC52A3
ABCC6	CLDN1	GABRA4	LDB3	PHYH	SLC5A1
ABCC8	CLDN10	GABRA5	LDHA	PHYKPL	SLC5A2
ABCC9	CLDN14	GABRA6	LDLR	PIEZO1	SLC5A4
ABCD1	CLDN16	GABRB1	LDLRAP1	PIEZO2	SLC5A5
ABCD3	CLDN19	GABRB2	LEMD3	PIGA	SLC5A7
ABCD4	CLEC7A	GABRB3	LEP	PIGH	SLC6A1
ABCG2	CLIC5	GABRD	LEPR	PIGL	SLC6A15
ABCG5	CLIP2	GABRE	LETM1	PIGM	SLC6A19
ABCG8	CLMP	GABRG1	LFNG	PIGN	SLC6A2
ABHD12	CLN3	GABRG2	LGI4	PIGO	SLC6A20
ABHD5	CLN5	GABRG3	LGSN	PIGT	SLC6A3
ABL1	CLN6	GABRP	LHB	PIGV	SLC6A4
ACACA	CLN8	GABRQ	LHCGR	PIGW	SLC6A8
ACACB	CLOCK	GABRR1	LHX1	PIGY	SLC6A9

Table S3 A list of 3472 DKD-related genes

Gene symbol	Gene symbol	Gene symbol	Gene symbol	Gene symbol	Gene symbol
ACAD8	CLP1	GABRR2	LHX3	PIK3CA	SLC7A1
ACAD9	CLRN1	GABRR3	LHX4	PIK3CG	SLC7A11
ACADL	CLU	GAD1	LIF	PIK3R1	SLC7A14
ACADM	CMA1	GAD2	LIFR	PIK3R2	SLC7A3
ACADS	CNBP	GALE	LIG4	PIK3R5	SLC7A4
ACADSB	CNDP1	GALK1	LIMK1	PIM1	SLC7A5
ACADVL	CNGA1	GALNS	LIMS2	PINK1	SLC7A7
ACAT1	CNGA3	GALNT3	LIN28B	PIP5K1C	SLC7A8
ACD	CNGB1	GALR1	LIPA	PITPNM3	SLC7A9
ACE	CNGB3	GALR2	LIPC	PITX2	SLC9A1
ACE2	CNNM2	GALR3	LIPE	PJVK	SLC9A3
ACER3	CNNM4	GALT	LIPN	PKD1	SLC9A3R1
ACHE	CNR1	GAMT	LIPT1	PKD2	SLC9A6
ACLY	CNR2	GAN	LMAN1	PKHD1	SLCO1A2
ACO2	CNTFR	GANAB	LMBRD1	PKLR	SLCO1B1
ACOX1	CNTNAP1	GARS	LMF1	PKP1	SLCO1B3
ACOX2	COA5	GAS1	LMNA	PKP2	SLCO2A1
ACP5	COA7	GAS6	LMNB1	PLA2G2A	SLITRK6
ACSL1	COASY	GATA1	LMNB2	PLA2G2E	SLX4
ACSL4	COCH	GATA2	LMO1	PLA2G4A	SMAD3
ACSS1	COG2	GATA3	LMOD1	PLA2G6	SMAD4
ACSS2	COG4	GATA4	LMOD3	PLA2G7	SMAD6
ACTA1	COG6	GATA5	LMX1B	PLAA	SMARCA2
ACTA2	COG7	GATA6	LONP1	PLAGL1	SMARCA4
ACTB	COG8	GATAD1	LOR	PLAT	SMARCAL1
ACTC1	COL10A1	GATB	LOX	PLAU	SMARCB1
ACTG1	COL11A1	GBA	LPA	PLAUR	SMARCD2
ACTG2	COL11A2	GBA2	LPIN1	PLCD1	SMARCE1
ACTN2	COL12A1	GBE1	LPIN2	PLCE1	SMC1A
ACTN4	COL13A1	GCDH	LPL	PLCG2	SMC3
ACVR1	COL17A1	GCG	LRAT	PLD1	SMCHD1
ACVRL1	COL18A1	GCGR	LRBA	PLD2	SMG9
ACY1	COL1A1	GCH1	LRIG2	PLD3	SMN1
ADA	COL1A2	GCK	LRIT3	PLEC	SMPD1
ADA2	COL25A1	GCLC	LRP1	PLEKHG4	SMPX
ADAM8	COL2A1	GCLM	LRP2	PLEKHG5	SMS
ADAM9	COL3A1	GCM2	LRP4	PLEKHM1	SNAI2

Table S3 A list of 3472 DKD-related genes

Gene symbol	Gene symbol	Gene symbol	Gene symbol	Gene symbol	Gene symbol
ADAMTS13	COL4A1	GCSH	LRP5	PLG	SNAP25
ADAMTS2	COL4A3	GDAP1	LRPAP1	PLIN1	SNAP29
ADAMTS3	COL4A4	GDF2	LRPPRC	PLK4	SNCA
ADAMTSL4	COL4A5	GDF3	LRRC8A	PLN	SNCAIP
ADCY1	COL4A6	GDF5	LRRK2	PLOD1	SNCB
ADCY10	COL5A1	GDF6	LSS	PLOD2	SNORD115-1
ADCY2	COL5A2	GDNF	LTB4R	PLP1	SNORD1161
ADCY6	COL6A1	GDPD3	LTB4R2	PLPBP	SNRNP200
ADCYAP1	COL6A2	GEMIN4	LTBP2	PLS3	SNRPN
ADGRE2	COL6A3	GFAP	LTBP3	PLTP	SNTA1
ADGRG2	COL7A1	GFER	LTBP4	PLXND1	SNX10
ADGRV1	COL9A1	GFPT1	LTF	PMM2	SNX14
ADH1A	COL9A2	GFPT2	LYRM7	PMP22	SOD1
ADH1B	COL9A3	GGCX	LYST	PMPCA	SOD2
ADH1C	COLQ	GH1	LYVE1	PMS1	SON
ADH4	COMT	GHR	LYZ	PMS2	SORD
ADH5	COQ2	GHRHR	LZTFL1	PMVK	SORL1
ADH6	COQ6	GHRL	LZTR1	PNKD	SOS1
ADH7	COQ7	GHSR	MAD2L2	PNKP	SOS2
ADIPOQ	COQ8A	GIF	MAF	PNLIP	SOST
ADK	COQ8B	GIG18	MAFA	PNP	SOX10
ADM	COX1	GIGYF2	MAFB	PNPLA1	SOX11
ADNP	COX10	GIP	MAG	PNPLA2	SOX17
ADORA1	COX14	GIPR	MAGED2	PNPLA6	SOX18
ADORA2A	COX15	GJA1	MAGEL2	PNPLA8	SOX2
ADORA2B	COX2	GJB1	MAGI2	PNPO	SOX3
ADRA1A	COX20	GJB2	MAK	PNPT1	SOX5
ADRA1B	COX3	GJB3	MALT1	POC1A	SOX9
ADRA1D	COX4I2	GJB4	MAN1B1	POC1B	SP110
ADRA2A	COX6B1	GJB6	MAN2B1	PODXL	SP7
ADRA2B	COX7B	GJC2	MANF	POF1B	SPARC
ADRA2C	COX8A	GK	MAOA	POGLUT1	SPART
ADRB1	CP	GLA	MAOB	POGZ	SPAST
ADRB2	CPA1	GLB1	MAP2	POLA1	SPATA5
ADRB3	CPB2	GLDC	MAP2K1	POLD1	SPATA7
ADSL	CPE	GLE1	MAP2K2	POLG	SPECC1L

Table S3 A list of 3472 DKD-related genes

Gene symbol	Gene symbol	Gene symbol	Gene symbol	Gene symbol	Gene symbol
AEBP1	CPLANE1	GLI2	MAP3K1	POLG2	SPG11
AFF4	CPLX1	GLI3	MAP3K20	POLR1A	SPG21
AFG3L2	CPOX	GLIS2	MAP3K5	POLR1C	SPG7
AGA	CPQ	GLIS3	MAP3K7	POLR1D	SPHK1
AGBL5	CPS1	GLP1R	MAP4	POLR3A	SPIB
AGGF1	CPT1A	GLP2R	MAPK1	POLR3B	SPIDR
AGK	CPT1B	GLRA1	MAPK10	POMC	SPINK1
AGL	CPT1C	GLRA2	MAPK12	POMGNT1	SPINK5
AGPAT1	CPT2	GLRX5	MAPK14	POMGNT2	SPN
AGPAT2	CR2	GLS	MAPK3	POMK	SPR
AGRN	CRAT	GLS2	MAPK8	POMT1	SPRTN
AGRP	CRB1	GLUD1	MAPK9	POMT2	SPRY4
AGT	CRB2	GLUD2	MAPKAPK 3	PON1	SPTBN2
AGTR1	CREB1	GLUL	MAPKBP1	PON2	SPTLC1
AGTR2	CREBBP	GLYCTK	MAPRE2	PON3	SPTLC2
AGXT	CRH	GM2A	MAPT	POR	SQLE
AGXT2	CRHBP	GMNN	MARS	PORCN	SQSTM1
AHI1	CRHR1	GMPPA	MARS2	POT1	SRA1
AHR	CRHR1IT1 CRHR1	GMPPB	MASP1	POU1F1	SRC
AHSG	CRHR2	GMPS	MASP2	POU2AF1	SRCAP
AIFM1	CRIPT	GNA1	MATR3	POU6F2	SRD5A1
AIMP1	CRK	GNAI2	MAX	PPARA	SRD5A2
AIP	CRKL	GNAL	MBD5	PPARD	SREBF1
AIPL1	CRLF1	GNAQ	MBTPS1	PPARG	SRP54
AIRE	CRP	GNAS	MBTPS2	PPARGC1A	SRY
AK2	CRTAP	GNASAS1	MC1R	PPAT	SSR4
AK9	CRX	GNAT1	MC2R	PPCS	SSTR2
AKAP9	CRYAB	GNAT2	MC3R	PPIB	ST3GAL5
AKR1A1	CRYM	GNB1	MC4R	PPM1B	STAC3
AKR1B1	CRYZ	GNB3	MCAM	PPM1D	STAT1
AKR1D1	CSF2RA	GNB5	MCCC1	PPOX	STAT3
AKT1	CSF2RB	GNE	MCCC2	PPP1R15B	STAT4
AKT2	CSF3	GNPTAB	MCEE	PPP1R3A	STEAP3
ALAD	CSF3R	GNRH1	MCFD2	PPP2R1A	STIM1
ALAS2	CSNK2A1	GNRHR	MCHR1	PPP2R2B	STK11
ALB	CSPP1	GORAB	MCHR2	PPP2R5D	STN1

Table S3 A list of 3472 DKD-related genes

Gene symbol	Gene symbol	Gene symbol	Gene symbol	Gene symbol	Gene symbol
ALDH18A1	CSRP3	GOT1	MCM4	PPP3CA	STRA6
ALDH1A3	CST3	GOT2	MCM9	PPP3R1	STRADA
ALDH2	CSTA	GP1BA	MCOLN1	PPT1	STRC
ALDH3A2	CTBP1	GP1BB	MDH2	PPY	STS
ALDH4A1	CTC1	GP6	MDM2	PRCC	STT3A
ALDH5A1	CTDP1	GP9	MECOM	PRCD	STT3B
ALDH7A1	CTF1	GPAA1	MECP2	PRDM16	STUB1
ALDOB	CTGF	GPBAR1	MECR	PRDM5	STX11
ALG1	CTLA4	GPC3	MED12	PRDM8	STX16
ALG11	CTNNA3	GPC4	MED25	PRDX1	STX3
ALG12	CTNNB1	GPC6	MEFV	PREP	STXBP1
ALG13	CTNND1	GPD1	MEGF10	PREPL	STXBP2
ALG14	CTNND2	GPD1L	MEGF8	PRF1	SUCLA2
ALG2	CTNS	GPI	MEN1	PRICKLE1	SUCLG1
ALG3	CTPS1	GPIHBP1	MEOX1	PRIMPOL	SUFU
ALG6	CTRC	GPNMB	MERTK	PRKAA1	SUGCT
ALG8	CTSC	GPR101	MESP2	PRKAB1	SULT2B1
ALG9	CTSD	GPR119	MET	PRKAB2	SUMF1
ALK	CTSG	GPR143	MFAP5	PRKACA	SURF1
ALMS1	CTSH	GPR161	MFF	PRKACG	SUZ12
ALOX12B	CTSK	GPR179	MFN2	PRKAG2	SYNE1
ALOX15	CUBN	GPR35	MFRP	PRKAR1A	SYNE2
ALOX5	CUL3	GPR39	MFSD8	PRKAR1B	SYNJ1
ALOXE3	CUL4B	GPR85	MGAM	PRKAR2B	SYT14
ALPI	CUL7	GPSM2	MGAT2	PRKCB	SYT2
ALPL	CUX1	GPT	MGLL	PRKCD	SYTL4
ALPP	CWF19L1	GPT2	MGME1	PRKCG	TAAR1
ALPPL2	CXCL8	GPX1	MGMT	PRKCSH	TAB2
ALS2	CXCR1	GPX4	MGP	PRKDC	TAC1
ALX3	CXCR2	GREB1L	MICU1	PRKG1	TAC3
ALX4	CYB5A	GREM1	MID1	PRKN	TACO1
AMACR	CYB5R3	GRHL2	MIF	PRKRA	TACR2
AMMECR1	CYBA	GRHPR	MINPP1	PRLR	TACR3
AMN	CYBB	GRIA1	MIR184	PRMT7	TACSTD2
AMPD1	CYLD	GRIA2	MIR204	PRNP	TAF1
AMPD2	CYP11A1	GRIA3	MIR96	PROC	TAF15
AMPD3	CYP11B1	GRIA4	MITF	PRODH	TAF1A
AMT	CYP11B2	GRID1	MKKS	PROK2	TAGLN

Table S3 A list of 3472 DKD-related genes

Gene symbol	Gene symbol	Gene symbol	Gene symbol	Gene symbol	Gene symbol
AMY2A	CYP17A1	GRID2	MKRN1	PROKR2	TALDO1
ANG	CYP19A1	GRIK1	MKRN3	PROM1	TANGO2
ANGPTL3	CYP1A1	GRIK2	MKRN3AS1	PROP1	TAP1
ANGPTL6	CYP1A2	GRIK3	MKS1	PROS1	TAP2
ANK1	CYP1B1	GRIK4	MLH1	PRPF3	TAPBP
ANK2	CYP21A2	GRIK5	MLH3	PRPF31	TAPT1
ANKH	CYP24A1	GRIN1	MLNR	PRPF4	TARDBP
ANKLE2	CYP26A1	GRIN2A	MLX	PRPF6	TARS
ANKRD1	CYP26C1	GRIN2B	MLXIPL	PRPF8	TARS2
ANKRD11	CYP27A1	GRIN2C	MLYCD	PRPH	TAS1R2
ANKRD55	CYP27B1	GRIN2D	MMAA	PRPH2	TAS1R3
ANKS6	CYP2A6	GRIN3A	MMAB	PRPS1	TAT
ANLN	CYP2B	GRIN3B	MMACHC	PRRT2	TAZ
ANO10	CYP2B6	GRIP1	MMADHC	PRSS1	TBC1D1
ANO3	CYP2C19	GRK1	MME	PRSS12	TBC1D20
ANO5	CYP2C8	GRK2	MMEL1	PRSS2	TBC1D23
ANOS1	CYP2C9	GRM1	MMP1	PRTN3	TBC1D24
ANTXR1	CYP2D6	GRM2	MMP12	PRUNE1	TBCD
ANTXR2	CYP2E1	GRM3	MMP13	PRX	TBCE
ANXA11	CYP2R1	GRM4	MMP19	PSAP	TBK1
AOC1	CYP2U1	GRM5	MMP2	PSAT1	TBL2
AP1S1	CYP3A4	GRM6	MMP8	PSEN1	TBP
AP1S2	CYP3A5	GRM7	MMP9	PSEN2	TBX1
AP2S1	CYP3A7	GRM8	MNX1	PSMB8	TBX15
AP3B1	CYP4A11	GRN	MOCOS	PSMC3IP	TBX18
AP3B2	CYP4F22	GSK3A	MOG	PSTPIP1	TBX19
AP3D1	CYP4V2	GSN	MOGS	PTCH1	TBX22
AP5Z1	CYP7A1	GSR	MORC2	PTCH2	TBX3
APC	CYP7B1	GSTM1	MPC1	PTDSS1	TBX6
APC2	CYSLTR2	GSTP1	MPDU1	PTEN	TBXA2R
APOA1	CYTB	GSTT1	MPDZ	PTF1A	TBXAS1
APOA5	D2HGDH	GTF2I	MPI	PTGDR2	TCAP
APOB	DAB1	GTF2IRD1	MPIG6B	PTGER4	TCF3
APOC2	DACT1	GTPBP2	MPL	PTGIR	TCF4
APOC3	DAG1	GTPBP3	MPO	PTGIS	TCIRG1
APOE	DAO	GUCA1A	MPV17	PTGS1	TCN1

Table S3 A list of 3472 DKD-related genes

Gene symbol	Gene symbol	Gene symbol	Gene symbol	Gene symbol	Gene symbol
APOPT1	DAOA	GUCA1B	MPZ	PTGS2	TCN2
APP	DARS2	GUCY1A1	MRAP	PTH	TCOF1
APRT	DAXX	GUCY1B3	MRGPRX2	PTH1R	TCTN3
APTX	DBH	GUCY2D	MRPS16	PTH2R	TDGF1
AQP2	DBT	GUSB	MRPS22	PTHLH	TDO2
AQP5	DCAF17	GYG1	MRPS34	PTK2B	TDP1
AR	DCAF8	GYS1	MRPS7	PTPN1	TECRL
ARAF	DCANP1	GYS2	MS4A1	PTPN11	TECTA
ARG2	DCC	GZF1	MSH2	PTPN14	TEK
ARHGAP31	DCDC2	H19	MSH6	PTPN2	TENM3
ARHGDIA	DCHS1	H19ICR	MSMO1	PTPN22	TERC
ARHGEF18	DCLRE1C	H6PD	MSN	PTPN3	TERF2IP
ARHGEF6	DCN	HABP2	MST1	PTPRJ	TERT
ARID1A	DCPS	HACE1	MSTN	PTPRO	TET2
ARID1B	DCTN1	HADH	MSTO1	PTPRS	TF
ARID2	DDAH1	HADHA	MTND1	PTRH2	TFAM
ARL13B	DDAH2	HADHB	MT1A	PTS	TFAP2A
ARL2BP	DDB2	HAMP	MT2A	PUS1	TFG
ARL6	DDC	HARS	MTAP	PWAR1	TFPI
ARMC5	DDHD1	HARS2	MTFMT	PWRN1	TFR2
ARNT2	DDHD2	HAVCR1	MTHFD1	PYCR1	TFRC
ARPC1B	DDIT4	HBA1	MTHFR	PYCR2	TG
ARSA	DDOST	HBA2	MTM1	PYGL	TGFB1
ARSE	DDR2	HBB	MTMR14	PYGM	TGFB2
ARTN	DDX3X	HBD	MTMR2	PYY	TGFB3
ARV1	DEAF1	HBG1	MTO1	QARS	TGFBI
ARVCF	DEFB4B	HBG2	MTOR	QDPR	TGFBR1
ARX	DES	HCAR1	MTR	QRICH1	TGFBR2
ASAH1	DGAT1	HCAR2	MTRR	RAB11B	TGFBR3
ASCC1	DGAT2	HCCS	MTTP	RAB18	TGIF1
ASCL1	DGCR2	HCN4	MUC1	RAB23	TGM1
ASIC1	DGCR6	HCRT	MUSK	RAB27A	TGM2
ASIC3	DGCR8	HCRTR1	MUT	RAB28	TGM3
ASL	DGKE	HDAC4	MVD	RAB33B	TGM4
ASNS	DGUOK	HDAC8	MVK	RAB39B	TGM5
ASPA	DHCR24	HELLS	MYBPC1	RAB3GAP1	TGM6

Table S3 A list of 3472 DKD-related genes

Gene symbol	Gene symbol	Gene symbol	Gene symbol	Gene symbol	Gene symbol
ASS	DHCR7	HEPACAM	MYBPC3	RAB3GAP2	TGM7
ASS1	DHDDS	HERC2	MYC	RAB40AL	TH
ASXL1	DHFR	HES7	MYCN	RAB7A	THAP1
ASXL2	DHH	HESX1	MYD88	RAC1	THBD
ATAD1	DHODH	HEXB	MYF6	RAC2	THNSL1
ATAD3A	DHTKD1	HEXIM1	MYH11	RAD21	THOC2
ATF6	DIABLO	HFE	MYH14	RAD51	THOC6
ATL1	DIAPH1	HGD	MYH2	RAD51C	THPO
ATL3	DIAPH3	HGF	MYH3	RAF1	THRA
ATM	DIS3L2	HGSNAT	MYH6	RAG1	THRB
ATOH7	DISC1	HIC1	MYH7	RAG2	TIMM50
ATP13A2	DISP1	HIF1A	MYH9	RAI1	TIMM8A
ATP1A1	DKC1	HIKESHI	MYL2	RALBP1	TIMMDC1
ATP1A2	DKK1	HINT1	MYL3	RAP1A	TINF2
ATP1A3	DLD	HIRA	MYLK	RAP1B	TJP2
ATP2A1	DLG3	HIVEP2	MYLK2	RAPSN	TK2
ATP2A2	DLG4	HJV	MYMK	RARA	TKT
ATP2B3	DLL1	HK1	MYO1A	RARB	TLR4
ATP2C1	DLL3	HLAA	MYO1E	RARG	TMC1
ATP4A	DLL4	HLAB	MYO5A	RASA2	TMC6
ATP5D	DLX5	HLADPB1	MYO5B	RAX2	TMC8
ATP5F1E	DLX6	HLADQA1	MYO7A	RB1	TMCO1
ATP6	DMD	HLADQB1	MYO9A	RBCK1	TMEM106B
ATP6AP1	DMP1	HLADRA	MYOC	RBM10	TMEM107
ATP6AP2	DMPK	HLADRB1	MYOT	RBM20	TMEM126A
ATP6V0A2	DMRT1	HLCS	MYOZ2	RBM28	TMEM126B
ATP6V0A4	DMRT3	HMBS	MYPN	RBM8A	TMEM127
ATP6V1A	DMXL2	HMGA2	MYT1L	RBMX	TMEM138
ATP6V1B1	DNA2	HMGCL	NAA10	RBP1	TMEM165
ATP6V1B2	DNAAF4	HMGCR	NAALAD2	RBP3	TMEM173
ATP6V1E1	DNAH1	HMGCS2	NADK2	RBP4	TMEM199
ATP7A	DNAJB11	HMOX1	NADSYN1	RBPJ	TMEM216
ATP7B	DNAJB2	HNF1A	NAGA	RCBTB1	TMEM231
ATP8	DNAJB6	HNF1B	NAGLU	RDH12	TMEM237
ATP8A2	DNAJC12	HNF4A	NAGS	RECQL4	TMEM240
ATP8B1	DNAJC13	HNRNPA1	NALCN	REEP1	TMEM260

Table S3 A list of 3472 DKD-related genes

Gene symbol	Gene symbol	Gene symbol	Gene symbol	Gene symbol	Gene symbol
ATXN2	DNAJC6	HOGA1	NBEAL2	RERE	TMP1
ATXN3	DNAL4	HOXA1	NBN	REST	TMPO
ATXN7	DNASE1L3	HOXA11	NCF1	RET	TMPRSS15
ATXN8	DNM1L	HOXA13	NCF2	RETREG1	TMPRSS3
ATXN8OS	DNM2	HOXB1	NCF4	REV3L	TMPRSS6
AUH	DNMT1	HOXD13	ND1	RFC2	TNF
AVP	DNMT3B	HP	ND2	RFT1	TNFRSF11A
AVPR1A	DNPEP	HPD	ND4	RFWD2	TNFRSF11B
AVPR2	DOCK6	HPGD	ND4L	RFWD3	TNFRSF13B
AXL	DOCK8	HPRT1	ND5	RFX5	TNFRSF13C
AZIN2	DOK7	HPS1	ND6	RFX6	TNFRSF1A
B2M	DOLK	HPS3	NDE1	RFXANK	TNFRSF1B
B3GALNT2	DPAGT1	HPS4	NDN	RFXAP	TNFRSF4
B3GALT6	DPF2	HPS5	NDP	RGR	TNFRSF9
B3GAT3	DPH1	HPS6	NDUFA1	RGS9	TNFSF11
B3GLCT	DPM1	HPSE2	NDUFA10	RGS9BP	TNFSF12
B4GALNT1	DPM3	HRAS	NDUFA11	RHBDF2	TNFSF15
B4GALT1	DPP4	HRG	NDUFA12	RHD	TNFSF4
B4GALT7	DPP6	HRH1	NDUFA13	RHO	TNNC1
B4GAT1	DPYD	HRH2	NDUFA2	RHOBTB2	TNNC2
BAAT	DPYS	HRH4	NDUFA3	RIMS1	TNNI2
BAG3	DRAM2	HS6ST1	NDUFA4	RIN2	TNNI3
BAK1	DRD1	HSD11B1	NDUFA5	RIPK4	TNNT2
BANF1	DRD2	HSD11B2	NDUFA6	RIPPLY2	TNNT3
BAP1	DRD3	HSD17B10	NDUFA7	RIT1	TNPO3
BAZ1B	DRD4	HSD17B3	NDUFA8	RLBP1	TNXB
BBIP1	DRD5	HSD17B4	NDUFA9	RMND1	TOMM40
BBS1	DSC2	HSD3B2	NDUFAF1	RMRP	TOP2A
BBS10	DSG2	HSD3B7	NDUFAF2	RNASE1	TOPORS
BBS12	DSG4	HSPA1A	NDUFAF3	RNASEH1	TOR1AIP1
BBS2	DSP	HSPA1B	NDUFAF4	RNASEH2A	TP53
BBS4	DSPP	HSPA9	NDUFAF5	RNASET2	TP53RK
BBS5	DSTYK	HSPB1	NDUFAF6	RNF125	TP63
BBS7	DTNA	HSPB3	NDUFB11	RNF168	TPH1
BBS9	DTNBP1	HSPB8	NDUFB3	RNF213	TPH2
BCAP31	DUOX2	HSPD1	NDUFB9	RNF216	TPI1

Table S3 A list of 3472 DKD-related genes

Gene symbol	Gene symbol	Gene symbol	Gene symbol	Gene symbol	Gene symbol
BCAT1	DUOXA2	HSPG2	NDUFS1	RNR1	TPM1
BCAT2	DUSP1	HTR1A	NDUFS2	RNU4ATAC	TPM2
BCHE	DUSP6	HTR1B	NDUFS3	ROBO1	TPM3
BCKDHA	DUX4	HTR1D	NDUFS4	ROBO3	TPO
BCKDHB	DVL1	HTR1E	NDUFS6	ROCK1	TPP1
BCL10	DVL3	HTR2A	NDUFS7	ROCK2	TPP2
BCL11B	DYNC1H1	HTR2B	NDUFS8	ROM1	TPRKB
BCL2	DYNC2H1	HTR2C	NDUFV1	ROR2	TPRN
BCL6	DYNC2LI1	HTR3A	NDUFV2	RP1	TPT1
BCOR	DYRK1B	HTR3B	NEB	RP1L1	TRAF3IP1
BCR	DYSF	HTR3C	NEBL	RP2	TRAF3IP2
BCS1L	DZIP1L	HTR3D	NECTIN1	RP9	TRAIP
BDKRB1	E	HTR3E	NEFH	RPE65	TRAPPC11
BDKRB2	EARS2	HTR4	NEK1	RPGR	TRAPPC9
BDNF	EBP	HTR6	NEK2	RPGRIP1	TRDN
BEAN1	ECE1	HTR7	NEK8	RPGRIP1L	TREM2
BEST1	ECHS1	HTRA1	NEK9	RPL10	TREX1
BET1	ECM1	HTRA2	NELFA	RPL11	TRH
BIN1	EDA	HTT	NELL1	RPL15	TRHR
BIRC3	EDA2R	HUWE1	NEU1	RPL18	TRIM2
BLK	EDARADD	HYAL1	NEUROG3	RPL26	TRIM28
BLM	EDC3	HYLS1	NEXMIF	RPL27	TRIM32
BLNK	EDN1	HYMAI	NEXN	RPL35	TRIM44
BLOC1S3	EDN3	IARS	NF1	RPL35A	TRIOBP
BMP1	EDNRA	IARS2	NF2	RPL5	TRIP11
BMP15	EDNRB	IBA57	NFIA	RPS10	TRIP13
BMP2	EED	ICAM1	NFIX	RPS17	TRIP4
BMP4	EEF1A2	ICK	NFKB1	RPS19	TRMT10A
BMPER	EFEMP1	ICOS	NFKB2	RPS24	TRMT5
BMPR1A	EFEMP2	IDE	NFS1	RPS26	TRNC
BMPR1B	EFHC1	IDH1	NFU1	RPS27	TRNE
BMPR2	EFL1	IDH2	NGF	RPS28	TRNF
BMS1	EFNB1	IDH3B	NGLY1	RPS29	TRNH
BOLA3	EGF	IDS	NHEJ1	RPS6KA3	TRNI
BRAF	EGFR	IDUA	NHLRC1	RPS7	TRNK
BRAT1	EGLN2	IER3IP1	NHP2	RPSA	TRNL1

Table S3 A list of 3472 DKD-related genes

Gene symbol	Gene symbol	Gene symbol	Gene symbol	Gene symbol	Gene symbol
BRCA1	EGR1	IFIH1	NHS	RRAS	TRNN
BRCA2	EGR2	IFNA2	NIPA1	RREB1	TRNP
BRIP1	EHHADH	IFNAR1	NIPAL4	RRM2B	TRNQ
BRS3	EHMT1	IFNAR2	NIPBL	RS1	TRNS1
BSCL2	EIF2AK3	IFNB1	NKX21	RSPO1	TRNS2
BSND	EIF2B1	IFNGR1	NKX25	RTEL1	TRNT1
BTD	EIF2B2	IFNGR2	NLRC4	RTN2	TRNV
BTK	EIF2B3	IFRD1	NLRP1	RTN4	TRNW
BTNL2	EIF2B4	IFT122	NLRP12	RTN4IP1	TRPA1
BTRC	EIF2B5	IFT140	NLRP3	RUBCN	TRPC3
BUB1	EIF2S3	IFT172	NLRP7	RUNX1	TRPC6
BUB1B	EIF4G1	IFT27	NME1	RUNX2	TRPM1
BUB3	ELANE	IFT43	NMNAT1	RXRA	TRPM4
BVES	ELMO2	IFT52	NNT	RXRB	TRPM6
C10orf10	ELN	IFT57	NOD2	RXRG	TRPM8
C12ORF65	ELOVL4	IFT74	NODAL	RXYLT1	TRPS1
C15ORF41	ELOVL5	IFT80	NOG	RYR1	TRPV1
C19ORF12	ELP1	IFT81	NOP10	RYR2	TRPV3
C19ORF70	ELP4	IFT88	NOP56	S100A1	TRPV4
C1QA	EMD	IGBP1	NOS1	S100A12	TSC1
C1QB	EMG1	IGF1	NOS2	S100A13	TSC2
C1QBP	ENG	IGF1R	NOS3	S100A2	TSEN2
C1QC	ENPEP	IGF2	NOTCH1	S100B	TSEN34
C1QTNF5	ENPP1	IGFALS	NOTCH2	S1PR2	TSEN54
C1R	ENTPD1	IGFBP3	NOTCH3	SAA1	TSFM
C1S	EOGT	IGFBP7	NOV	SACS	TSHB
C2	EP300	IGH	NPAP1	SAG	TSHR
C3	EPCAM	IGHM	NPC1	SALL1	TSPAN12
C4A	EPG5	IGHMBP2	NPC1L1	SALL2	TSPEAR
C5AR1	EPHA4	IGKC	NPC2	SALL4	TSPO
C8ORF37	EPHB4	IGLL1	NPHP1	SAMD9	TSPYL1
C9ORF72	EPHX1	IGSF1	NPHP3	SAMD9L	TSR2
CA1	EPHX2	IGSF3	NPHP4	SAMHD1	TTBK2
CA12	EPM2A	IKBKB	NPHS1	SAR1B	TTC19
CA14	EPO	IKBKE	NPHS2	SARS2	TTC21B
CA2	EPOR	IKBKG	NPPA	SBDS	TTC37
CA4	EPRS	IKZF1	NPPB	SBF1	TTC7A

Table S3 A list of 3472 DKD-related genes

Gene symbol	Gene symbol	Gene symbol	Gene symbol	Gene symbol	Gene symbol
CA5A	EPS15L1	IL10	NPR1	SBF2	TTC8
CA8	ERAP1	IL12A	NPR2	SC5D	TTLL5
CABP4	ERBB3	IL12AAS1	NPR3	SCAP	TTN
CACNA1A	ERBB4	IL12B	NPY	SCAPER	TTPA
CACNA1B	ERCC1	IL12RB1	NPY1R	SCARB1	TTR
CACNA1C	ERCC2	IL13	NPY2R	SCARB2	TUB
CACNA1D	ERCC3	IL17F	NPY5R	SCD	TUB1
CACNA1E	ERCC4	IL17RA	NQO1	SCGB1A1	TUB2
CACNA1F	ERCC5	IL17RC	NR0B1	SCN10A	TUBA3D
CACNA1G	ERCC6	IL17RD	NR0B2	SCN11A	TUBB
CACNA1H	ERCC6L2	IL18RAP	NR1H2	SCN1A	TUBB1
CACNA1S	ERCC8	IL1A	NR1H3	SCN1B	TUBB2B
CACNA2D1	EREG	IL1B	NR1H4	SCN2A	TUBB3
CACNA2D2	ERG11	IL1R2	NR1I2	SCN2B	TUBB4A
CACNA2D3	ERGIC1	IL1RN	NR1I3	SCN3A	TUBB4B
CACNA2D4	ERLIN1	IL21R	NR2E3	SCN4A	TUBGCP4
CACNB1	ERLIN2	IL23R	NR2F1	SCN4B	TUBGCP6
CACNB2	ERMARD	IL2RA	NR3C1	SCN5A	TUFM
CACNB4	ESCO2	IL2RB	NR3C2	SCN8A	TULP1
CACNG1	ESPN	IL2RG	NR4A2	SCN9A	TWIST1
CACNG2	ESR1	IL31	NR5A1	SCNN1A	TWIST2
CAD	ESR2	IL31RA	NRAS	SCNN1B	TWNK
CALCRL	ESRRA	IL36RN	NRL	SCNN1G	TXN2
CALM1	ESRRB	IL3RA	NRTN	SCO1	TXNL4A
CALM2	ESS2	IL5	NRXN1	SCP2	TXNRD2
CALM3	ETFA	IL6	NSD1	SCTR	TYMP
CALR	ETFB	IL7	NSD2	SCYL1	TYR
CAMK2B	ETFDH	IL7R	NSDHL	SDCCAG8	TYROBP
CAMKMT	ETHE1	ILDR1	NSMCE2	SDHA	UBAC2
CAMTA1	ETV6	IMPDH1	NSMF	SDHAF1	UBE2A
CANT1	EWSR1	IMPG2	NSUN2	SDHAF2	UBE2T
CAPN1	EXOSC2	INF2	NT5C3A	SDHB	UBE3A
CAPN3	EXOSC3	INPP5D	NT5E	SDHC	UBE3B
CAPN5	EXOSC8	INPP5E	NTF3	SDHD	UBQLN2
CARD11	EXT1	INPP5K	NTN1	SDR9C7	UBR1
CARD14	EXT2	INPPL1	NTRK1	SEC23B	UBTF
CARS2	EYA1	INS	NTRK2	SEC24C	UCHL1

Table S3 A list of 3472 DKD-related genes

Gene symbol	Gene symbol	Gene symbol	Gene symbol	Gene symbol	Gene symbol
CASK	EYA4	INSR	NUBPL	SEC61A1	UCP1
CASP1	EYS	INTU	NUP107	SEC63	UCP2
CASP10	EZH2	INVS	NUP205	SELE	UCP3
CASQ1	F	IPW	NUP93	SELENON	UFC1
CASQ2	F10	IQCB1	NUS1	SELL	UFD1
CASR	F11	IQSEC2	NXN	SELP	UFSP2
CAT	F12	IRAK4	NYX	SEM1	UGT2B15
CATSPER2	F13A1	IRF3	OAT	SEMA3A	UGT2B7
CAV1	F13B	IRF5	OBSL1	SEMA3C	UMOD
CAV3	F2	IRF6	OCA2	SEMA3D	UMPS
CAVIN1	F2R	IRF8	OCLN	SEMA3E	UNC119
CBL	F2RL1	IRS1	OCRL	SEMA4A	UNC13A
CBS	F3	IRX5	OFD1	SEMA5A	UNC13B
CBX2	F5	ISCA2	OGDH	SERAC1	UNC13D
CC2D2A	F8	ISCU	OPA1	SERPINA1	UNC80
CCBE1	F9	ISL1	OPA3	SERPINA3	UPB1
CCBL1	FA2H	ISPD	OPLAH	SERPINA6	UQCC2
CCBL2	FAAH	ITGA1	OPN1LW	SERPINC1	UQCC3
CCDC115	FABP2	ITGA2	OPN1MW	SERPIND1	UQCRB
CCDC141	FABP4	ITGA2B	OPRD1	SERPINE1	UQCRC2
CCDC22	FABP5	ITGA3	OPRK1	SERPING1	UROD
CCDC50	FAH	ITGA4	OPRL1	SETBP1	UROS
CCDC78	FAM111A	ITGA5	OPRM1	SETD2	USB1
CCDC8	FAM111B	ITGA6	OPTN	SETD5	USH1C
CCDC88A	FAM126A	ITGA7	ORAI1	SETX	USH1G
CCDC88C	FAM161A	ITGA8	ORC1	SF3B1	USH2A
CCK	FAM20A	ITGAE	ORC4	SF3B4	USP18
CCKAR	FAM20C	ITGAV	ORC6	SFRP4	USP8
CCL2	FAN1	ITGB1	ORM1	SFTPB	USP9X
CCM2	FANCA	ITGB2	OSBPL2	SFTPC	UTP4
CCND1	FANCB	ITGB3	OSGEP	SFXN4	UTS2
CCNF	FANCC	ITGB4	OSMR	SGCA	VAC14
CCNQ	FANCD2	ITGB5	OTC	SGCB	VAMP1
CCR1	FANCE	ITK	OTOA	SGCD	VAMP7
CCR6	FANCF	ITM2B	OTOF	SGCG	VANGL1
CCT5	FANCG	ITPA	OTULIN	SGPL1	VANGL2
CD14	FANCI	ITPR1	OTX2	SH2B1	VAPB

Table S3 A list of 3472 DKD-related genes

Gene symbol	Gene symbol	Gene symbol	Gene symbol	Gene symbol	Gene symbol
CD151	FANCL	IVD	OXCT1	SH2B3	VARS
CD160	FANCM	IYD	OXT	SH2D1A	VCAM1
CD163	FAP	JAG1	P2RX2	SH3BP2	VCL
CD164	FARS2	JAK1	P2RX3	SH3PXD2B	VCP
CD19	FARSB	JAK2	P2RX4	SH3TC2	VDR
CD1A	FAS	JMJD1C	P2RX7	SHANK3	VEGFA
CD247	FASLG	JPH1	P2RY1	SHH	VEGFC
CD27	FASTKD2	JPH2	P2RY11	SHOX	VHL
CD28	FAT4	JPH3	P2RY12	SHPK	VIPAS39
CD2AP	FBLN5	JRK	P3H2	SIGMAR1	VIPR2
CD36	FBN1	JUN	PABPN1	SIK1	VKORC1
CD40LG	FBN2	JUP	PACS1	SIL1	VLDLR
CD55	FBP1	KANK2	PACS2	SIM1	VMA21
CD58	FBXL4	KANSL1	PADI1	SIN3A	VPS11
CD59	FBXO38	KAT6A	PADI2	SIRT1	VPS13A
CD6	FBXO7	KAT6B	PADI3	SIRT2	VPS13B
CD79A	FCER2	KBTBD13	PADI4	SIX1	VPS13C
CD79B	FCGR1A	KCNA1	PADI6	SIX3	VPS33A
CD81	FCGR2A	KCNA5	PAFAH1B1	SIX5	VPS33B
CD9	FCGR2B	KCNAB2	PAH	SIX6	VPS35
CD96	FCGR2C	KCNC3	PALB2	SKI	VPS37A
CDC42	FCGR3A	KCND2	PALLD	SKIV2L	VPS45
CDC45	FCGR3B	KCND3	PAM16	SLC10A1	VPS53
CDC6	FDFT1	KCNE1	PANK2	SLC10A2	VRK1
CDC73	FDPS	KCNE2	PARK2	SLC11A1	VWA3B
CDCA7	FDXR	KCNE3	PARN	SLC11A2	VWF
CDH1	FECH	KCNE5	PARP1	SLC12A1	WAC
CDH23	FERMT1	KCNH2	PAX2	SLC12A2	WARS2
CDH3	FERMT3	KCNJ1	PAX3	SLC12A3	WAS
CDHR1	FEZF1	KCNJ10	PAX4	SLC12A4	WASHC5
CDK10	FFAR1	KCNJ11	PAX6	SLC12A5	WDPCP
CDK4	FFAR2	KCNJ13	PAX8	SLC12A6	WDR11
CDK5	FGA	KCNJ18	PBX1	SLC13A5	WDR19
CDK5RAP2	FGB	KCNJ2	PC	SLC15A1	WDR26
CDK9	FGD1	KCNJ3	PCARE	SLC15A2	WDR34
CDKL5	FGD4	KCNJ5	PCCA	SLC16A1	WDR35

Table S3 A list of 3472 DKD-related genes

Gene symbol	Gene symbol	Gene symbol	Gene symbol	Gene symbol	Gene symbol
CDKN1A	FGF10	KCNJ6	PCCB	SLC16A10	WDR45
CDKN1B	FGF12	KCNJ8	PCDH15	SLC16A12	WDR60
CDKN1C	FGF14	KCNJ9	PCDH19	SLC16A2	WDR73
CDKN2A	FGF17	KCNK3	PCK1	SLC16A3	WDR81
CDKN2B	FGF20	KCNN4	PCK2	SLC16A7	WFS1
CDKN2C	FGF21	KCNQ1	PCLO	SLC17A5	WHRN
CDKN2D	FGF23	KCNQ1OT1	PCNA	SLC17A8	WIPF1
CDON	FGF3	KCNQ2	PCNT	SLC17A9	WIPI1
CDSN	FGF8	KCNQ3	PCSK1	SLC18A1	WISP3
CDT1	FGFR1	KCNQ4	PCSK2	SLC18A2	WNK1
CEACAM1	FGFR2	KCNQ5	PCSK9	SLC18A3	WNK4
CEBPB	FGFR3	KCNT1	PCYT1A	SLC19A2	WNT1
CEL	FGFR4	KCTD1	PCYT1B	SLC19A3	WNT10B
CENPF	FGFRL1	KDM1A	PDCD10	SLC1A1	WNT3A
CENPJ	FGG	KDM6A	PDE10A	SLC1A2	WNT4
CEP120	FH	KDR	PDE11A	SLC1A3	WNT5A
CEP126	FHL1	KDSR	PDE1A	SLC1A4	WRAP53
CEP164	FHL2	KHK	PDE1B	SLC1A5	WRN
CEP19	FIBP	KIAA0319L	PDE3A	SLC1A6	WT1
CEP290	FIG4	KIAA0586	PDE4A	SLC1A7	WWOX
CEP41	FKBP10	KIAA1109	PDE4B	SLC20A2	XDH
CEP57	FKBP14	KIDINS220	PDE4D	SLC22A1	XIAP
CEP78	FKBP4	KIF11	PDE5A	SLC22A11	XK
CEP83	FKBP5	KIF1A	PDE6A	SLC22A12	XPA
CEPT1	FKRP	KIF1B	PDE6B	SLC22A2	XPC
CERKL	FKTN	KIF1C	PDE6C	SLC22A3	XPNPEP3
CERS3	FLAD1	KIF23	PDE6G	SLC22A4	XPR1
CES1	FLCN	KIF5A	PDE6H	SLC22A5	XRCC1
CETP	FLG2	KIF7	PDE7A	SLC22A6	XRCC2
CFAP410	FLI1	KISS1	PDE7B	SLC22A7	XRCC4
CFB	FLII	KISS1R	PDE8B	SLC22A8	XYLT1
CFH	FLNA	KIT	PDGFB	SLC24A1	XYLT2
CFHR5	FLNB	KITLG	PDGFD	SLC24A5	YAP1
CFI	FLNC	KIZ	PDGFRA	SLC25A1	YARS
CFL2	FLRT3	KL	PDGFRB	SLC25A13	YARS2
CFLAR	FLT1	KLC2	PDHA1	SLC25A15	YWHAE

Table S3 A list of 3472 DKD-related genes

Gene symbol	Gene symbol	Gene symbol	Gene symbol	Gene symbol	Gene symbol
CFTR	FLT4	KLF1	PDHX	SLC25A18	YWHAG
CH17360D	FLVCR1	KLF11	PDK2	SLC25A19	YY1AP1
CHAT	FMO3	KLF13	PDK3	SLC25A20	ZAP70
CHCHD10	FMR1	KLHL3	PDLIM5	SLC25A22	ZBTB20
CHD2	FN1	KLHL40	PDPK1	SLC25A24	ZBTB24
CHD4	FN3K	KLHL41	PDSS1	SLC25A26	ZDHHC15
CHD7	FNDC4	KLHL7	PDSS2	SLC25A38	ZEB1
CHDH	FOLH1	KLK2	PDX1	SLC25A4	ZEB2
CHEK2	FOS	KLLN	PDXK	SLC25A46	ZFP57
CHKA	FOXC1	KLRC4	PDYN	SLC26A1	ZFPM2
CHKB	FOXC2	KMT2A	PDZD7	SLC26A2	ZFYVE26
CHM	FOXE1	KMT2D	PEMT	SLC26A3	ZFYVE27
CHMP2B	FOXE3	KNG1	PEPD	SLC26A4	ZIC1
CHN1	FOXF1	KRAS	PET100	SLC26A5	ZIC2
CHRDL1	FOXG1	KRIT1	PEX1	SLC29A1	ZIC3
CHRM1	FOXH1	KRT1	PEX10	SLC29A2	ZMPSTE24
CHRM2	FOXI1	KRT10	PEX11B	SLC29A3	ZNF202
CHRM3	FOXP1	KRT12	PEX12	SLC2A1	ZNF365
CHRM4	FOXP3	KRT13	PEX13	SLC2A10	ZNF408
CHRM5	FOXRED1	KRT14	PEX14	SLC2A2	ZNF423
CHRNA1	FPGS	KRT18	PEX16	SLC30A10	ZNF469
CHRNA10	FRA16E	KRT2	PEX19	SLC30A9	ZNF513
CHRNA2	FRAS1	KRT3	PEX2	SLC31A1	ZNF592
CHRNA3	FREM1	KRT5	PEX26	SLC33A1	ZNF687
CHRNA4	FREM2	KRT6A	PEX3	SLC34A1	ZNHIT3
CHRNA5	FRG1	KRT8	PEX5	SLC34A3	ZSWIM6
CHRNA6	FRMD4A	KY	PEX6	SLC35A1	
CHRNA7	FRMD7	KYNU	PEX7	SLC35C1	
CHRNA9	FSCN2	L1CAM	PF4	SLC35D1	

Table S4 Enrichment results of clinical symptoms of 390 CRT putative targets

Symptom Name	Putative Target	Gene Count	P value
Pollakiuria	CHRND,CHRNB1,CHRNB3,CHRNB2,CHRNB4,CHRNA10,CHRNA3,CHRNA4,CHRNA5,CHRNA6,CHRNA7,CHRNA1,CHRNA2,CHRNE,CHRNA9,CHRNG	16	9.91E-08
Diabetic Neuropathy	CHRND,CHRNE,CHRNB1,CHRNB3,CHRNB2,CHRNB4,CHRNA10,CHRNA4,CHRNA5,CHRNA6,CHRNA7,HTR2A,CHRNA1,CHRNA2,CHRNA3,CHRNA9,CHRNG	17	1.16E-07
Inflammation	PTGS2,TLR4,ADORA2A,ADORA1,MPO,PPARG,CASP1,PRKCE,CHUK,ADAM8,ADK,IKBKB,IFNG,PPARD,PLA2G1B,IL1B,ADRB2,SERPINE1	18	0.0000181
Hypokalemia	NR3C1,TP53,CACNA1D,AIP,SLC2A2,CYP17A1,ADRB2,COL5A1,SCN4A,CYP11B2,CACNA1S,HSD11B2	12	0.000316
Hypertensive Disease	PTGS2,NCF1,NOS2,PPARG,CYBA,ACE2,AGTR1,HSD11B2,HSD11B1	9	0.000808
Hypertension	NCF1,TGFB3,ACE,NR3C2,CACNA1D,SMAD6,TGFB2,CYP17A1,B2M,PDE11A,CACNA1H,CBS,COX2,COX3,COX1,PDE3A,ADRA2A,ADRA2B,CACNA1G,SCN2B,HSD11B2,TGFBR2,NR3C1,ENG,REST,ENPP1,CYBB,PPARG,COL5A1,CACNA2D1,PDE8B,TP53,ADRA1D,CALR,GCH1,NOTCH2,ADRB1,ADRB2,ADRB3,AIP,CYP11B2,AGTR1	42	0.00162

Table S4 Enrichment results of clinical symptoms of 390 CRT putative targets

Symptom Name	Putative Target	Gene Count	P value
Decreased Circulating Aldosterone Level	NR3C2,NR3C1,NR0B1,HSD3B2,CYP11B2,HSD11B2	6	0.00209
Diabetic Retinopathy	PRKCB,VEGFA,SERPINE1,CASP1	4	0.0031
Decreased Activity Of Nadph Oxidase	CYBB,NCF2,NCF1,CYBA	4	0.0031
Hyperkalemia	NR3C2,RYR1,SLC2A1,HSD3B2,SCN4A,CYP11B2,CACNA1S,GABRA3	8	0.00408
Postprandial Hyperglycemia	SCN4A,INSR,CACNA1S,GABRA3	4	0.0219
Renal Cell Carcinoma	TGFB1,TP53,HDAC4,PTGS2,PTEN,PIK3CA,PPARG,MET,AKT1,MMP9,FOLR1,TSC1	12	0.023
Decreased Circulating Renin Level	NR3C2,HSD11B2,CACNA1D,CYP17A1	4	0.0326
Hypotension	ADORA2A,NR3C2,RYR1,DRD2,ALB,AIP,HSD3B2,HRH1,DDC,CYP11B2	10	0.0447
Diabetic Nephropathy	AOC1,HTR2A,ACE,JAK1	4	0.0455
Urinary Retention	TGFB1,CACNA1S,PRNP,GABRA3	4	0.0455

Table S4 Enrichment results of clinical symptoms of 390 CRT putative targets

Symptom Name	Putative Target	Gene Count	P value
Episodic Hypokalemia	SCN4A,CACNA1S,GABRA3	3	0.0469

Table S5 Enrichment results of functional modules of 390 CRT putative targets

Function module	Pathway	Genes	Count	%	PValue
Substance metabolism, energy synthesis and decomposition, as well as hormone synthesis and metabolism	hsa04930:Type II diabetes mellitus	PRKCZ, PIK3CB, PIK3CD, SOCS1, PRKCE, PRKCD, SLC2A2, PKLR, CACNA1G, PIK3CA, MTOR, CACNA1C, IKBKB, CACNA1D, INSR, CACNA1A, CACNA1B	17	2.74	6.68E-07
	hsa05211:Renal cell carcinoma	BRAF, PIK3CB, PIK3CD, MET, EGLN3, TGFB3, EGLN2, EGLN1, TGFB1, TGFB2, AKT1, HIF1A, JUN, VEGFA, SLC2A1, RAC1, PIK3CA	17	2.74	1.78E-04
	hsa00670:One carbon pool by folate	SHMT1, SHMT2, ALDH1L1, AMT, FTCD, MTHFD1L, GART, MTHFD1, TYMS, MTHFS, MTHFD2, MTHFR, DHFR, ATIC, MTR, MTFMT	16	2.58	9.47E-16
	hsa00230:Purine metabol ism	ENPP1, PDE11A, PDE3B, ADA, PDE6A, PDE6B, PDE6C, PDE1B, ATIC, NT5M, PDE1C, PDE4A, PDE4B, NT5C2, PDE1A, PDE8B, PDE8A, IMPDH1, IMPDH2, PDE10A, PDE4D, PDE3A, PDE4C, GART, PDE7B, PDE2A, PDE7A, ADK, PKLR, PDE5A, PDE9A, RDH14	32	5.15	2.85E-06
	hsa00260:Glycine, serine and threonine metabolism	SHMT1, SHMT2, AMT, PSPH, GLDC, CTH, SDS, BHMT, PHGDH, SRR, AGXT2, PSAT1, CBS	13	2.09	3.66E-06
	hsa00140:Steroid hormone biosynthesis	CYP3A4, HSD3B2, AKR1C2, CYP17A1, HSD3B1, HSD17B1, CYP11B2, HSD11B1, SRD5A1, SRD5A2, AKR1D1, AKR1C1, CYP19A1	13	2.09	3.12E-04
	hsa00830:Retinol metabolism	CYP3A4, ALDH1A1, RDH12, RDH8, RDH11, ALDH1A2, DHRS3, DHRS4, LRAT, ADH1C, RETSAT, RDH5	12	1.93	0.004727 18
	hsa00410:beta-Alanine m etabolism	ACADM, EHHADH, ABAT, DPYS, HIBCH, DPYD, ALDH9A1	7	1.07	0.010374 916

Table S5 Enrichment results of functional modules of 390 CRT putative targets

Function module	Pathway	Genes	Count	%	PValue
Nervous system regulation	hsa04080:Neuroactive ligand-receptor interaction	OPRM1, TSPO, ADORA3, GLRA1, GABRB3, GABRB2, TRPV1, GRIK2, GABRB1, GLRA3, LHCGR, GLRA2, GRIN3B, GNRHR, GRIN3A, ADORA1, AGTR1, PTGIR, HTR1B, HTR1A, GRIN2B, GRIN2C, GRIN2D, HTR1D, GABRG1, GABRG2, PTGER1, GABRG3, PTGER2, PTGER3, PTGER4, HTR4, GRIN2A, CHRM5, ADRB2, ADRB1, CHRM4, CHRM3, CHRM2, CHRM1, GRM7, HTR6, DRD1, DRD3, ADORA2B, ADORA2A, DRD2, OPRK1, DRD5, DRD4, NR3C1, ADRB3, HRH1, HRH2, CNR1, CNR2, HRH4, ADRA2A, ADRA2C, ADRA2B, GABRQ, GABRP, GABRD, GABRE, GLRB, GABRA2, GABRA1, GABRA4, GABRA3, GABRA6, GABRA5, GRIN1, PTGFR, PLG, PRLR, GRIA2, MLNR, MTNR1B, ADRA1B, ADRA1A, HTR2B, HTR2C, ADRA1D, MTNR1A, HTR2A, OPRD1	86	13.85	1.71E-31
	hsa04020:Calcium signaling pathway	LHCGR, AGTR1, GRIN2C, GRIN2D, CHRNA7, NOS2, CHRFAM7A, PRKCA, PTGER1, PTGER3, GRIN2A, HTR4, PRKCG, PRKCB, ADRB2, CHRM5, ADRB1, CHRM3, CHRM2, CHRM1, HTR6, RYR1, DRD1, ADORA2B, ADORA2A, ERBB2, DRD5, PPP3R2, ADRB3, HRH1, PDE1B, HRH2, PDE1C, PDE1A, SLC8A1, CACNA1I, GRIN1, ITPR3, PTGFR, CACNA1S, ITPR1, ITPR2, ADRA1B, CACNA1G, CACNA1H, ADRA1A, CACNA1F, HTR2B, CACNA1C, HTR2C, CACNA1D, ADRA1D, CACNA1A, CACNA1B, HTR2A	54	8.70	1.49E-17

Table S5 Enrichment results of functional modules of 390 CRT putative targets

Function module	Pathway	Genes	Count	%	PValue
Nervous system regulation	hsa04540:Gap junction	PRKCA, DRD1, DRD2, PRKCG, ITPR3, ITPR1, ITPR2, PRKCB, TUBA8, TUBB, ADRB1, TUBA3C, TUBA4A, TUBA3D, TUBA3E, MAPK7, TUBA1A, HTR2B, HTR2C, TUBA1B, TUBA1C, HTR2A	21	3.38	3.65E-05
	hsa04720:Long-term potentiation	PRKCA, GRIN2B, GRIA2, BRAF, GRIN2C, GRIN2D, GRIN1, GRIN2A, PPP3R2, PRKCG, CACNA1C, ITPR3, ITPR1, ITPR2, PRKCB	15	2.42	0.001353 608
	hsa04730:Long-term depression	PRKCA, PPP2R1A, BRAF, PRKCG, ITPR3, ITPR1, ITPR2, PRKCB, GRIA2, PPP2CA, PPP2CB, PLA2G1B, RYR1, PLA2G2E, CACNA1A	15	2.42	0.001570 307
Cardiovascular system regulation	hsa04270:Vascular smooth muscle contraction	PRKCA, ADORA2B, BRAF, ADORA2A, PRKCG, ITPR3, PRKCE, CACNA1S, PRKCD, ITPR1, ITPR2, PRKCB, AGTR1, PRKCQ, PTGIR, ADRA1B, PLA2G1B, ADRA1A, CACNA1F, CACNA1C, PLA2G2E, CACNA1D, ADRA1D	23	3.70	1.32E-04
	hsa04960:Aldosterone-regulated sodium reabsorption	PRKCA, PIK3CB, PIK3CD, HSD11B1, ATP1A3, NR3C2, PIK3CA, PRKCG, ATP1A1, ATP1A2, INSR, PRKCB	12	1.93	4.25E-04
	hsa04370:VEGF signaling pathway	PRKCA, PTGS2, PIK3CB, PIK3CD, PPP3R2, PRKCG, PRKCB, AKT1, RAC2, RAC3, VEGFA, RAC1, PLA2G1B, HSPB1, PIK3CA, PLA2G2E	16	2.58	0.001260 093
Immune system regulation	hsa04670:Leukocyte transendothelial migration	PRKCA, ACTB, PIK3CG, ITGAL, NCF2, NCF1, PIK3CB, MMP9, NCF4, NOX1, PIK3CD, PRKCG, ITGB2, PRKCB, CYBA, CYBB, RAC2, PTK2B, RAC1, PIK3CA	20	3.05	0.007450 058

Table S5 Enrichment results of functional modules of 390 CRT putative targets

Function module	Pathway	Genes	Count	%	PValue
Immune system regulation	hsa04664:Fc epsilon RI signaling pathway	PRKCA, IL4, PIK3CG, PIK3CB, PIK3CD, PRKCE, PRKCD, PRKCB, AKT1, RAC2, RAC3, RAC1, PLA2G1B, PIK3CA, PLA2G2E	15	2.29	0.007966 851
Cell function regulation	hsa04010:MAPK signaling pathway	FGF18, FGF16, TGFB3, CACNB1, PPP3R2, CACNB2, CACNB3, CACNB4, TGFB1, TGFB2, AKT1, CASP3, RAC2, HSPA2, RAC3, MAPT, RAC1, PLA2G1B, IL1B, FGF1, FGF2, CHUK, FGF4, PRKCA, CACNA2D1, BRAF, CACNA1I, TGFBR2, TP53, PRKCG, CACNG1, CACNA2D3, CACNA2D2, CACNA1S, PRKCB, JUN, CACNA1G, CACNA1H, HSPB1, CACNA1F, MAPK7, IKBKB, PLA2G2E, CACNA1C, CACNA1D, CACNA1A, CACNA1B	47	7.18	6.10E-06
	hsa04150:mTOR signaling pathway	PIK3CG, AKT1, HIF1A, TSC1, BRAF, PIK3CB, PIK3CD, VEGFA, PIK3CA, MTOR, RICTOR, RPTOR	12	1.83	0.005104 611

Table S6 A list of 231 candidate targets of CRT against DKD in “Disease Gene-Drug Target” interaction networks

Node	Degree	Betweenness Centrality	Closeness Centrality	Node	Degree	Betweenness Centrality	Closeness Centrality
ACAT1	16	1.97E-04	0.33	MTNR1A	12	9.26E-05	0.32
ACE	8	1.14E-04	0.32	MTOR	62	0.0024776	0.38
ACSL3	10	4.06E-04	0.36	NCF1	38	0.00162336	0.37
ACTB	138	0.01577423	0.43	NCF2	14	7.61E-05	0.33
ACY1	8	1.77E-04	0.32	NCOA1	54	4.45E-04	0.37
ADRA1B	9	4.79E-04	0.32	NCOA2	51	0.0025451	0.37
ADRB2	126	0.01073076	0.39	NF2	14	8.66E-05	0.35
AGTR1	11	3.96E-04	0.33	NKX3-1	11	2.32E-04	0.33
AHR	20	1.06E-04	0.36	NOS2	52	0.00108134	0.37
AIP	21	9.78E-04	0.35	NOTCH2	17	3.04E-04	0.33
AKAP1	10	3.00E-04	0.34	NR1I2	19	1.34E-04	0.34
AKAP6	15	2.57E-04	0.35	NR3C1	87	0.00377042	0.40
AKT1	198	0.02187513	0.44	NR3C2	14	1.27E-04	0.34
ALB	75	0.01208351	0.37	OPRD1	12	1.44E-04	0.33
ALDH5A1	8	7.16E-05	0.33	OPTN	34	0.00110968	0.35
ANXA1	41	0.00142517	0.38	P3H1	13	0.00198886	0.33
APBB1	28	6.39E-04	0.37	P4HA1	11	1.97E-04	0.34
APOE	27	0.00165705	0.35	PAM	8	9.63E-05	0.32
AR	138	0.00784701	0.42	PDE3A	11	1.10E-04	0.33
ATIC	22	8.98E-04	0.34	PDE4D	16	1.51E-04	0.36
ATM	79	0.00317469	0.39	PDHB	19	2.51E-04	0.36
ATP1A1	30	0.00178826	0.39	PGR	25	1.13E-04	0.36

Table S6 A list of 231 candidate targets of CRT against DKD in "Disease Gene-Drug Target" interaction networks

Node	Degree	Betweenness Centrality	Closeness Centrality	Node	Degree	Betweenness Centrality	Closeness Centrality
ATP5B	46	0.0014383	0.38	PHYH	8	2.06E-04	0.32
ATP5C1	27	0.00118199	0.38	PIK3CA	40	0.00112352	0.36
BCL2	52	0.00221403	0.39	PIK3CB	17	1.96E-04	0.34
BRAF	32	3.01E-04	0.36	PIK3CG	27	3.45E-04	0.33
BRF1	8	1.81E-04	0.34	PIM1	28	8.33E-04	0.37
CACNA1A	32	0.00168922	0.34	PINK1	23	1.39E-04	0.35
CACNA1C	16	3.86E-04	0.35	PKM	48	0.0014698	0.40
CAD	48	8.28E-04	0.38	PKP2	20	1.86E-04	0.35
CALR	48	0.00427455	0.38	PLAT	18	3.26E-04	0.34
CAMLG	8	8.62E-04	0.32	PLG	37	0.00208053	0.36
CASP1	29	0.0014902	0.34	PLOD2	14	4.85E-04	0.33
CASP3	64	0.00224347	0.39	PLOD3	14	1.93E-04	0.35
CAV3	15	3.37E-04	0.32	PPARA	26	5.87E-04	0.35
CBR1	12	1.08E-04	0.35	PPARD	22	1.97E-04	0.35
CDK6	44	0.00136678	0.36	PPARG	58	0.00169502	0.39
CDKN1A	82	0.00583148	0.40	PPIA	35	7.25E-04	0.38
CEBPA	51	0.00100933	0.37	PPIF	9	5.43E-05	0.33
CEBPB	43	6.38E-04	0.38	PPP2CA	81	0.00544168	0.40
CHD8	13	7.66E-04	0.33	PPP2CB	40	9.05E-04	0.36
CHUK	79	0.00197702	0.40	PPP2R1A	63	0.00227034	0.40
COX6C	8	7.00E-04	0.32	PRDX4	17	0.00142951	0.35
CPS1	16	5.49E-04	0.33	PRDX5	9	8.10E-05	0.35

Table S6 A list of 231 candidate targets of CRT against DKD in "Disease Gene-Drug Target" interaction networks

Node	Degree	Betweenness Centrality	Closeness Centrality	Node	Degree	Betweenness Centrality	Closeness Centrality
CSNK2A1	182	0.02269164	0.43	PRKCB	56	0.00455894	0.37
CSNK2B	85	0.01058711	0.40	PRKCD	128	0.00869133	0.42
CYBA	12	0.00138691	0.34	PRKCE	55	0.00128487	0.38
CYCS	13	7.94E-04	0.33	PRKCG	27	6.33E-04	0.36
CYP17A1	10	6.26E-04	0.33	PRKCI	31	0.00108155	0.37
DIAPH1	14	3.26E-04	0.33	PRKCQ	28	2.50E-04	0.34
DLG4	49	0.00492445	0.37	PRKCZ	69	0.00112284	0.39
DNMT1	30	2.77E-04	0.36	PRKD1	32	8.60E-04	0.38
DRD2	15	2.12E-04	0.32	PRKD2	18	4.02E-04	0.34
DYRK2	28	3.31E-04	0.37	PRKDC	112	0.00701656	0.41
EGLN1	12	8.42E-05	0.35	PRNP	25	0.00135956	0.36
EHHADH	10	1.51E-04	0.33	PSAT1	8	1.73E-04	0.34
EIF3F	25	8.44E-04	0.37	PTEN	50	0.00260911	0.37
ELL	9	1.49E-04	0.32	PTGS2	12	1.38E-04	0.34
ERBB2	102	0.00562506	0.39	PTK2B	60	0.00222002	0.38
ESR1	241	0.01972767	0.44	PTPN2	16	2.89E-04	0.35
ESR2	129	0.01041908	0.39	RAC1	74	0.00659885	0.39
ESRRA	20	1.81E-04	0.34	RARA	69	0.0025005	0.39
ESRRG	18	2.29E-04	0.35	RARB	19	1.11E-04	0.34
F12	8	1.66E-04	0.33	RARG	17	1.01E-04	0.34
FASN	28	2.24E-04	0.37	RBP1	8	7.07E-04	0.33
FBLN1	20	0.00130266	0.35	RDX	19	2.50E-04	0.34

Table S6 A list of 231 candidate targets of CRT against DKD in "Disease Gene-Drug Target" interaction networks

Node	Degree	Betweenness Centrality	Closeness Centrality	Node	Degree	Betweenness Centrality	Closeness Centrality
FGF2	20	0.00164542	0.34	RIPK1	40	4.17E-04	0.36
FKBP1A	26	6.67E-04	0.35	RIPK3	60	0.00121864	0.37
GART	12	1.46E-04	0.34	RPL23A	58	6.38E-04	0.39
GRIA2	15	2.86E-04	0.32	RPTOR	28	3.48E-04	0.35
GRIK2	16	0.00109474	0.34	RUVBL2	82	0.00326003	0.41
GRIN1	35	9.22E-04	0.35	RXRA	71	0.00325875	0.38
GRIN2A	14	1.93E-04	0.34	RXRB	35	6.30E-04	0.34
GRIN2B	17	1.69E-04	0.34	RXRG	20	5.97E-05	0.33
GRIN2D	17	5.72E-05	0.34	RYR1	15	9.46E-05	0.33
GRM7	19	1.08E-04	0.33	S100A1	18	2.32E-04	0.34
GSK3B	156	0.01347391	0.43	S100A9	20	1.20E-04	0.34
GSN	23	4.24E-04	0.38	S100B	16	0.00132953	0.33
GSS	13	6.88E-05	0.32	SCN5A	12	1.02E-04	0.32
H2AFY	19	3.15E-04	0.37	SF3B3	36	3.56E-04	0.37
HCK	46	0.00107942	0.38	SHANK3	9	8.05E-05	0.34
HDAC1	196	0.01796659	0.42	SHBG	21	6.04E-04	0.33
HDAC2	131	0.00676118	0.40	SHMT1	11	6.76E-05	0.33
HDAC4	86	0.00697172	0.39	SHMT2	17	2.33E-04	0.36
HDAC5	129	0.00540061	0.40	SLC2A1	10	5.55E-05	0.32
HDAC9	35	7.10E-04	0.35	SMAD2	125	0.01061039	0.40
HIF1A	59	0.00141983	0.39	SMAD7	40	6.35E-04	0.37
HIPK2	32	4.12E-04	0.35	SOCS1	39	4.21E-04	0.36

Table S6 A list of 231 candidate targets of CRT against DKD in "Disease Gene-Drug Target" interaction networks

Node	Degree	Betweenness Centrality	Closeness Centrality	Node	Degree	Betweenness Centrality	Closeness Centrality
HSPA2	25	2.70E-04	0.38	TELO2	15	1.49E-04	0.35
HSPB1	104	0.01058598	0.41	TGFB1	34	0.00182369	0.36
HTR2A	11	1.14E-04	0.32	TGFB2	15	3.33E-04	0.33
IKBKB	89	0.00387834	0.40	TGFBR2	49	0.00132535	0.38
IL1B	10	7.77E-04	0.32	TLR4	20	4.90E-04	0.35
IMPDH2	22	4.89E-04	0.36	TOP1	47	0.00203244	0.39
INSR	53	0.00105352	0.37	TOP2A	36	4.73E-04	0.39
ITGB2	23	7.29E-04	0.35	TOP2B	25	1.29E-04	0.36
ITPR1	20	4.03E-04	0.35	TP53	245	0.02713821	0.44
ITPR3	13	1.19E-04	0.32	TSC1	25	4.79E-04	0.35
JAK1	55	0.00218823	0.37	TUBA1A	69	0.00252526	0.40
JUN	375	0.05297153	0.44	TUBA1B	45	8.40E-04	0.38
JUP	53	0.00169143	0.37	TUBA1C	54	0.00112181	0.38
KCNH2	15	4.75E-04	0.35	TUBA3C	24	9.04E-05	0.35
LARP1	28	2.04E-04	0.37	TUBA3D	24	9.04E-05	0.35
LPL	15	0.0016363	0.34	TUBA4A	46	0.00172142	0.38
MAP1A	15	3.94E-04	0.35	TUBB	100	0.00526006	0.42
MAP2	17	2.25E-04	0.36	TUBG1	44	0.00150006	0.37
MAP4	16	3.09E-04	0.35	TYMS	17	2.04E-04	0.33
MAPK7	29	0.00102826	0.37	UBA1	50	0.00311633	0.37
MAPKAP1	21	1.97E-04	0.35	UMPS	9	3.30E-04	0.33
MAPT	58	0.00196423	0.40	VDR	49	8.98E-04	0.39

Table S6 A list of 231 candidate targets of CRT against DKD in “Disease Gene-Drug Target” interaction networks

Node	Degree	Betweenness Centrality	Closeness Centrality	Node	Degree	Betweenness Centrality	Closeness Centrality
MTHFD1	22	2.73E-04	0.36				

Table S7 Enrichment results of functional modules of 231 CRT candidate targets

Function module	Pathway	Genes	Count	%	PValue
Renal basement membrane lesions	Focal adhesion	HRAS, PTEN, CTNNB1, VCL, AKT1, ACTG1, CDC42, PDPK1, PIK3CA, AKT2, PRKCA, EGFR, PIK3CG, BRAF, ROCK1, ACTN4, PIK3CB, PRKCG, ACTN2, FLNC, FLNB, FLNA, PRKCB, MAPK1, CCND1, CRKL, JUN, MAPK3, VEGFA, COL1A2, PDGFRA, MAPK9, PDGFRB, MAPK8, COL1A1, ITGA2B, CAV3, CAV1, XIAP, DIAPH1, ERBB2, ITGB4, ITGB5, ITGB3, ITGB1, SRC, IGF1R, SOS1, BCL2, ITGAV, SOS2, RAC1, COL6A2, PIK3R1, FN1, PIK3R2, ACTB, FLT1, COL4A1, MAP2K1, FLT4, MET, RAF1, IGF1, ITGA3, ITGA4, MAPK10, BIRC3, KDR, ITGA6, ITGA5, GSK3B, RAP1A, RAP1B, CRK, MYLK	76	6.76	6.70E-17
	MAPK signaling pathway	HRAS, FASLG, NFKB1, NFKB2, DAXX, TGFB1, TGFB2, AKT1, MAP3K7, CDC42, FOS, MAX, MAP3K5, BDNF, CASP3, MAPT, IL1B, PRKACA, FAS, FGF1, FGF2, MYC, CHUK, AKT2, PRKCA, EGFR, BRAF, TP53, PRKCG, CACNG2, FLNC, FLNB, FLNA, PRKCB, MAPK1, CRKL, JUN, MAPK3, PDGFRA, MAPK9, PDGFRB, HSPB1, MAPK8, MAPK7, FGFR2, FGFR1, FGFR4, TNF, FGFR3, MAPKAPK3, HSPA1A, PPM1B, HSPA1B, TNFRSF1A, KRAS, HSPA2, SOS1, MAP3K1, RAC1, SOS2, PPP3CA, MAP2K1, MAP2K2, TGFBR1, NF1, TGFBR2, RAF1, MAPK10, TAB2, NRAS, PLA2G4A, RPS6KA3, MAPK12, MAPK14, NTRK1, IKBKG, NTRK2, RAP1A, RAP1B, IKBKB, CACNA1C, CRK, CACNA1A	82	7.29	3.08E-12

Table S7 Enrichment results of functional modules of 231 CRT candidate targets

Function module	Pathway	Genes	Count	%	PValue
Renal basement membrane lesions	Regulation of actin cytoskeleton	HRAS, VCL, ACTG1, CDC42, GSN, PIK3CA, MSN, FGF1, FGF2, EGFR, PIK3CG, BRAF, ACTN4, ROCK1, LIMK1, PIK3CB, ARHGEF6, ACTN2, MYH9, WAS, MAPK1, ARPC1B, CRKL, MAPK3, F2, PDGFRA, PDGFRB, ITGA2B, FGFR2, FGFR1, FGFR4, FGFR3, DIAPH1, ITGB4, ITGB5, ITGB2, RDX, ITGB3, BDKRB2, ITGB1, PFN1, KRAS, INS, SOS1, ITGAV, SOS2, RAC1, PIK3R1, FN1, APC, PIK3R2, ACTB, MAP2K1, MAP2K2, RAF1, IGF2, ITGA3, ITGA4, NRAS, ITGA6, ITGA5, ARAF, MYH14, CRK, MYLK, SLC9A1	65	5.78	1.68E-09
	Apoptosis	TNF, XIAP, FASLG, NFKB1, AKT1, TNFRSF1A, CASP3, MYD88, BCL2, PIK3CA, IL1B, CSF2RB, PRKACA, PPP3CA, FAS, CHUK, PIK3R1, PIK3R2, AKT2, PIK3CG, CFLAR, AIFM1, PIK3CB, CYCS, TP53, BIRC3, ATM, CAPN1, CASP10, PRKAR1B, NTRK1, RIPK1, PRKAR1A, IKBKG, IKBKB	35	3.11	7.88E-09
	ErbB signaling pathway	HRAS, ERBB4, ERBB3, ERBB2, SRC, AKT1, KRAS, SOS1, GAB1, SOS2, PIK3CA, CAMK2B, MYC, PIK3R1, PIK3R2, AKT2, EGFR, PIK3CG, PRKCA, MAP2K1, BRAF, PIK3CB, MAP2K2, CBL, RAF1, PRKCG, MAPK10, PRKCB, MAPK1, NRAS, CDKN1A, CRKL, CDKN1B, GSK3B, JUN, ARAF, MAPK3, PLCG2, MAPK9, MAPK8, MTOR, ABL1, CRK	43	3.82	2.28E-14
	Adherens junction	FGFR1, ERBB2, CTNND1, CDH1, SRC, VCL, CTNNB1, ACTG1, MAP3K7, CDC42, IGF1R, CSNK2A1, RAC1, INSR, PTPRJ, EGFR, ACTB, ACTN4, TGFBR1, MET, TGFBR2, CREBBP, SMAD4, CSNK2B, SMAD3, SMAD2, ACTN2, WAS, MAPK1, EP300, MAPK3, PTPN1	32	2.84	1.59E-08

Table S7 Enrichment results of functional modules of 231 CRT candidate targets

Function module	Pathway	Genes	Count	%	PValue
Renal basement membrane lesions	mTOR signaling pathway	PIK3CG, BRAF, STK11, PIK3CB, IGF1, IGF2, RICTOR, RPTOR, AKT1, MAPK1, RPS6KA3, PDPK1, HIF1A, TSC1, INS, VEGFA, TSC2, MAPK3, PIK3CA, PRKAA1, MTOR, PIK3R1, AKT2, PIK3R2	23	2.04	7.23E-07
	Cell cycle	PRKDC, CHEK2, TGFB1, TGFB2, CDC45, CDKN2A, RAD21, BUB1, MYC, BUB3, CDC6, CREBBP, SMAD4, TP53, SMAD3, CDK6, SMAD2, RB1, ATR, CDK4, MCM4, YWHAE, SMC3, ATM, CDKN1A, CCND1, YWHAG, CDKN1B, HDAC2, EP300, HDAC1, GSK3B, PCNA, BUB1B, MDM2, ABL1, SMC1A	37	3.29	1.58E-05
	TGF-beta signaling pathway	PPP2R1A, ACVRL1, TNF, ROCK1, SMAD7, TGFBR1, CREBBP, TGFBR2, SMAD4, BMPR2, SMAD3, SMAD2, DCN, TGFB1, TGFB2, MAPK1, EP300, PPP2CA, PPP2CB, MAPK3, BMPR1B, MYC, ACVR1, BMPR1A, PITX2	25	2.22	8.12E-04
	Wnt signaling pathway	PPARD, PPP2R5D, BTRC, CTNNB1, MAP3K7, CHD8, CSNK2A1, PPP2CA, PPP2CB, RAC1, PRKACA, CAMK2B, PPP3CA, MYC, APC, PRKCA, DVL3, PPP2R1A, CTBP1, ROCK1, CREBBP, SMAD4, CSNK2B, TP53, SMAD3, PRKCG, SMAD2, MAPK10, PRKCB, DVL1, CCND1, EP300, PSEN1, GSK3B, JUN, MAPK9, MAPK8	37	3.29	9.98E-04
	p53 signaling pathway	CYCS, TP53, IGF1, CDK6, ATR, CHEK2, CDK4, PTEN, ATM, RFWD2, CCND1, CDKN1A, CASP3, CDKN2A, TSC2, DDB2, MDM2, FAS, IGFBP3	19	1.69	0.005696776
	hsa04622:RIG-I-like receptor signaling pathway	TNF, TBK1, NFKB1, MAPK10, MAP3K7, CASP10, IKBKE, CYLD, MAPK12, DDX3X, MAPK14, RIPK1, MAP3K1, IKBKG, MAPK9, IRF3, MAPK8, IKBKB, CHUK	19	1.69	0.009174711

Table S7 Enrichment results of functional modules of 231 CRT candidate targets

Function module	Pathway	Genes	Count	%	PValue
Immune-inflammatory response	B cell receptor signaling pathway	HRAS, NFKB1, BTK, AKT1, FOS, KRAS, SOS1, SOS2, RAC1, PIK3CA, PPP3CA, INPP5D, CHUK, PIK3R1, AKT2, BLNK, PIK3R2, PIK3CG, BCL10, MAP2K1, PIK3CB, MAP2K2, RAF1, PRKCB, CARD11, MAPK1, NRAS, CD19, FCGR2B, FCGR2C, GSK3B, JUN, CD81, PLCG2, IKBKG, MAPK3, IKBKB	36	3.20	1.22E-11
	T cell receptor signaling pathway	HRAS, TNF, CD247, NFKB1, MAP3K7, AKT1, CDC42, FOS, KRAS, SOS1, SOS2, ZAP70, PIK3CA, PPP3CA, CHUK, PIK3R1, AKT2, PIK3R2, PIK3CG, BCL10, ITK, MAP2K1, PIK3CB, MAP2K2, CBL, RAF1, CDK4, CARD11, MAPK1, LAT, PRKCQ, NRAS, MAPK12, CD40LG, MAPK14, GSK3B, JUN, LCK, IKBKG, MAPK3, MAPK9, IKBKB	42	3.73	6.24E-10
	Fc epsilon RI signaling pathway	HRAS, TNF, BTK, AKT1, KRAS, SOS1, SOS2, RAC1, PIK3CA, INPP5D, PIK3R1, PIK3R2, AKT2, PRKCA, PIK3CG, MAP2K1, PIK3CB, MAP2K2, RAF1, MAPK10, PRKCE, PRKCD, PRKCB, MAPK1, LAT, NRAS, PLA2G4A, MAPK12, MAPK14, PLCG2, MAPK3, MAPK9, MAPK8	33	2.93	5.32E-09
	Fc gamma R-mediated phagocytosis	AKT1, CDC42, GSN, RAC1, PIK3CA, INPP5D, FCGR3A, PIK3R1, PIK3R2, AKT2, PIK3CG, PRKCA, PLD2, PLD1, DNM1L, MAP2K1, LIMK1, PIK3CB, NCF1, HCK, RAF1, PRKCG, PRKCE, PRKCD, WAS, PRKCB, MAPK1, LAT, ARPC1B, PLA2G4A, CRKL, FCGR2B, FCGR2C, PLCG2, MAPK3, FCGR2A, CRK, DNM2	37	3.29	7.53E-09

Table S7 Enrichment results of functional modules of 231 CRT candidate targets

Function module	Pathway	Genes	Count	%	PValue
Immune-inflammatory response	Toll-like receptor signaling pathway	TNF, TBK1, TLR4, NFKB1, MAP3K7, AKT1, FOS, MYD88, RAC1, PIK3CA, IL1B, CHUK, PIK3R1, PIK3R2, AKT2, PIK3CG, MAP2K1, PIK3CB, MAP2K2, MAPK10, STAT1, TAB2, IFNAR1, MAPK1, IFNAR2, IKBKE, MAPK12, IRF5, MAPK14, JUN, RIPK1, IKBKG, MAPK3, MAPK9, MAPK8, IRF3, IKBKB	37	3.29	4.90E-08
	Natural killer cell mediated cytotoxicity	HRAS, TNF, CD247, FASLG, ITGB2, SH2D1A, CASP3, KRAS, PTK2B, SOS1, SOS2, RAC1, ZAP70, PIK3CA, PPP3CA, FAS, FCGR3A, IFNGR1, PIK3R1, PIK3R2, PIK3CG, PRKCA, ICAM1, MAP2K1, BRAF, PIK3CB, MAP2K2, HLA-A, RAF1, PRKCG, HLA-B, PRKCB, IFNAR1, PTPN11, MAPK1, LAT, IFNAR2, NRAS, ARAF, MAPK3, PLCG2, LCK	42	3.73	5.74E-07
	Leukocyte transendothelial migration	GNAI2, CTNND1, ITGB2, ITGB1, MMP2, VCL, CTNNB1, VCAM1, ACTG1, CDC42, PTK2B, RAC1, PIK3CA, MSN, PIK3R1, PIK3R2, PIK3CG, PRKCA, ACTB, ICAM1, ITK, ACTN4, NCF2, ROCK1, PIK3CB, NCF1, PRKCG, ACTN2, ITGA4, PRKCB, PTPN11, CYBA, MAPK12, MAPK14, PLCG2, RAP1A, RAP1B	37	3.29	3.71E-06
	NOD-like receptor signaling pathway	HSP90AA1, TNF, XIAP, NFKB1, MAPK10, NLRP3, BIRC3, TAB2, MAP3K7, MAPK1, NOD2, MAPK12, MAPK14, IKBKG, MAPK3, PSTPIP1, MAPK9, IL1B, MAPK8, CASP1, IKBKB, CHUK	22	1.96	7.51E-05
	Chemokine signaling pathway	PRKCZ, HRAS, GNAI2, NFKB1, CXCR2, AKT1, CDC42, KRAS, PTK2B, SOS1, SOS2, RAC1, PIK3CA, PRKACA, CHUK, PIK3R1, PIK3R2, AKT2, PIK3CG, ITK, MAP2K1, ROCK1, BRAF, PIK3CB, NCF1, HCK, RAF1, STAT1, PRKCD, WAS, STAT3, PRKCB, MAPK1, NRAS, CRKL, GSK3A, GNB1, GSK3B, MAPK3, IKBKG, RAP1A, JAK2, RAP1B, IKBKB, CRK, GRK1	46	4.09	1.93E-04

Table S7 Enrichment results of functional modules of 231 CRT candidate targets

Function module	Pathway	Genes	Count	%	PValue
Immune-inflammatory response	Primary immunodeficiency	CIITA, CD19, CD40LG, TAP2, LCK, IKBKG, TAP1, AIRE, ZAP70, IL7R, RFXANK, BTK, BLNK	13	1.16	0.002326823
	Neurotrophin signaling pathway	HRAS, FASLG, NFKB1, AKT1, CDC42, MAP3K5, BDNF, GAB1, PIK3CA, AKT2, PIK3CG, BRAF, PIK3CB, TP53, IRS1, PRKCD, MAPK1, CRKL, PSEN1, JUN, MAPK3, MAPK9, MAPK8, MAPK7, KRAS, BCL2, MAP3K1, SOS1, SOS2, RAC1, CAMK2B, ARHGDIA, PIK3R1, PIK3R2, MAP2K1, MAP2K2, RAF1, MAPK10, YWHAE, PTPN11, NRAS, YWHAG, RPS6KA3, MAPK12, MAPK14, GSK3B, NTRK1, NTRK2, PLCG2, RAP1A, CALM3, RAP1B, ABL1, IKBKB, CRK, CALM2, CALM1	55	4.89	1.09E-15
Nervous system	Long-term potentiation	HRAS, KRAS, GRIN2B, GRIN2D, PRKACA, CAMK2B, PPP3CA, PRKCA, MAP2K1, BRAF, MAP2K2, GRIN1, CREBBP, GRIN2A, RAF1, PRKCG, ITPR3, GRM1, ITPR1, PRKCB, MAPK1, NRAS, RPS6KA3, EP300, GRIA2, GNAQ, GRIA1, ARAF, MAPK3, CALM3, RAP1A, RAP1B, CACNA1C, CALM2, CALM1	33	2.93	7.21E-11
	Gap junction	HRAS, GNAI2, TUBB2B, DRD2, GJA1, PRKG1, SRC, TUBB, KRAS, MC1R, SOS1, SOS2, TUBA3C, TUBA3D, PRKACA, TUBB1, TUBA1A, TUBA1B, TUBA1C, TUBB3, EGFR, PRKCA, MAP2K1, MAP2K2, RAF1, PRKCG, ITPR3, GRM1, ITPR1, PRKCB, MAPK1, NRAS, GNAQ, MAPK3, PDGFRA, TUBA4A, PDGFRB, GNAS, MAPK7, HTR2A	38	3.38	2.09E-10

Table S7 Enrichment results of functional modules of 231 CRT candidate targets

Function module	Pathway	Genes	Count	%	PValue
	Long-term depression	HRAS, GNAI2, PRKG1, IGF1R, KRAS, PPP2CA, PPP2CB, PRKCA, PPP2R1A, NOS1, MAP2K1, BRAF, MAP2K2, IGF1, RAF1, PRKCG, ITPR3, GRM1, ITPR1, PRKCB, CRHR1, MAPK1, NRAS, PLA2G4A, GNAQ, GRIA2, GRIA1, ARAF, MAPK3, RYR1, GNAS, CACNA1A	32	2.84	6.18E-10
	Tight junction	PRKCZ, HRAS, GNAI2, CASK, PTEN, SRC, CTNNB1, ACTG1, AKT1, CDC42, CSNK2A1, KRAS, PPP2CA, PPP2CB, PPP2R2B, AKT2, PRKCA, ACTB, PPP2R1A, MAGI2, ACTN4, MPDZ, CSNK2B, PRKCI, PRKCG, ACTN2, MYH9, PRKCE, CDK4, PRKCD, PRKCB, NRAS, PRKCQ, MYH11, MYH14, TJP2	36	3.20	1.86E-04
	Calcium signaling pathway	ERBB4, ERBB3, ERBB2, BDKRB2, AGTR1, PTK2B, GRIN2D, NOS3, CAMK2B, PRKACA, PPP3CA, NOS2, EGFR, PRKCA, NOS1, SLC25A4, PHKG2, GRIN1, GRIN2A, PRKCG, ITPR3, GRM1, ITPR1, PRKCB, ADRB2, ATP2A2, GNAQ, HTR7, ATP2A1, PLCG2, ADRA1B, PDGFRA, RYR1, TBXA2R, CALM3, PDGFRB, RYR2, GNAS, CACNA1C, CALM2, CACNA1A, MYLK, CALM1, HTR2A	42	3.73	7.53E-04
Energy metabolism	Adipocytokine signaling pathway	PPARA, TNF, STK11, PRKAG2, NFKB1, POMC, AKT1, TNFRSF1A, TNFRSF1B, SLC2A1, PRKAA1, ACSL3, CHUK, AKT2, RXRB, RXRA, PRKAB2, RXRG, PRKAB1, MAPK10, IRS1, PPARGC1A, STAT3, CPT1A, PCK1, PTPN11, PRKCQ, CD36, IKBKG, MAPK9, MAPK8, JAK2, MTOR, IKBKB	34	3.02	7.41E-12
	Fatty acid elongation in mitochondria	ECHS1, PPT1, HADH, HADHA, HADHB	5	0.44	0.017894823

Table S7 Enrichment results of functional modules of 231 CRT candidate targets

Function module	Pathway	Genes	Count	%	PValue
Energy metabolism	Valine, leucine and isoleucine biosynthesis	IARS, LARS, PDHA1, IARS2, VARS, PDHB	6	0.53	0.012824922
	hsa00650:Butanoate metabolism	ALDH5A1, OXCT1, EHHADH, ALDH2, ECHS1, PDHA1, HADH, ACAT1, ALDH3A2, HADHA, PDHB	11	0.98	0.017081213
Abnormal renal hemorheology	VEGF signaling pathway	HRAS, PTGS2, MAPKAPK3, SRC, AKT1, CDC42, KRAS, RAC1, PIK3CA, NOS3, PPP3CA, PIK3R1, AKT2, PIK3R2, PRKCA, PIK3CG, MAP2K1, MAP2K2, PIK3CB, RAF1, PRKCG, KDR, PRKCB, MAPK1, NRAS, PLA2G4A, MAPK12, MAPK14, VEGFA, PLCG2, MAPK3, HSPB1	32	2.84	7.47E-09
	Aldosterone-regulated sodium reabsorption	PRKCA, PIK3CG, PIK3CB, NR3C2, IGF1, PRKCG, IGF2, ATP1A1, IRS1, PRKCB, MAPK1, PDPK1, KRAS, INS, MAPK3, PIK3CA, INSR, PIK3R1, PIK3R2	18	1.60	1.86E-05
	Vascular smooth muscle contraction	PRKG1, AGTR1, PRKACA, PRKCA, MAP2K1, ROCK1, BRAF, ACTA2, MAP2K2, NPR1, RAF1, PRKCG, NPR2, PRKCE, ITPR3, PRKCD, ITPR1, PRKCB, MAPK1, PRKCQ, PLA2G4A, GNAQ, ARAF, MAPK3, ADRA1B, MYH11, CALM3, GNAS, CACNA1C, CALM2, MYLK, CALM1	30	2.67	7.60E-04
	Complement and coagulation cascades	PLAT, KNG1, F12, MASP1, C3, F13A1, C1R, BDKRB2, PLG, C1QA, F5, FGA, FGB, CD59, F2, TFPI, CFH, SERPINA1, PLAU	19	1.69	0.00671007
